# Supplementary material for: The impact of environmental factors on the evolution of brain size in carnivorans
Source: Commun Biol. 2022 Sep 21;5:998. doi: 10.1038/s42003-022-03748-4 (PMC9492690; doi:10.1038/s42003-022-03748-4)
Supplement: Supplementary file 2 — Supplementary Information [file 42003_2022_3748_MOESM2_ESM.pdf]

Supplementary Note 1. Definition of diet, locomotion and activity pattern categories used in this study.

Diet :
Carnivorous : Diet including a significant portion of vertebrates
Frugivorous : Diet including a significant portion of fruits
Herbivorous and folivorous : Diet including a significant portion of leaves and /or grass
Invertivorous : Diet including a significant portion of arthropods and worms
Omnivorous : Diet consisting in animal prey, in addition to a variety of fruits or other vegetable matter
Piscivorous : Diet including a significant portion of fish

Locomotion:
Aquatic : Spend more time in the water than on the ground and can even sleep in water
Arboreal : Spend more time in the trees than on the ground and forage actively in trees
Semi-arboreal : Spend a significant part of their time in the trees
Semi-fossorial : Spend a significant part of their time on the ground and often dig to make a burrow or find food
Terrestrial : Spend a significant part of their time on the ground, but occasionally climb, swim or dig

Activity pattern :
Cathemeral or crepuscular : Animals with an irregularly active pattern at any time of day or night and animals more active at dawn
Diurnal : More active at day
Nocturnal : More active at night

Supplementary Note 2. Definition of social complexity categories.

Category 0: Obligatory solitary species (except for mating).
Category 1: Solitary specie but can stand with other (e.g. temporary coalition).
Category 2: Pair-living, the young quitting the family unit before the next litter.
Category 3: Pair-living and young staying in the family units for multiple litters.
Category 4: Group-living, not only close relatives, no hierarchical level.
Category 5: Group-living, not only close relatives, one hierarchical level (i.e. dominants versus others).
Category 6: Group-living, not only close relatives, multiple hierarchical levels.

Supplementary Figure 1. Analyses of the differences of means, variances and proportions of the predictors used in this study.

For categorical predictors (A-E), chi-squared contingency tables were used to test for proportions homogeneity of the different categories according to the taxonomic groups. Test result are provided at the bottom left of each graph. The proportion of each category is indicated as a percentage for values greater than 4%. Cells representing negative residuals - values smaller than expected from a normal distribution - are drawn in blue and with broken edges while positive values - values larger than expected from a normal distribution - are drawn in red with solid borders. For continuous predictors (F-M), Bartlett's test were performed to assess the homogeneity of variances at different taxonomic scales in parallel with ANOVA tests which provides information on the differences in means between these groups. Test results are provided to the right of each graph, below the legend box. Figures associated represent the density distribution of each predictor according to taxonomic groups with dotted lines representing the mean of each of the groups. P-values are shown for each significantly positive Tukey post-hoc pairwise test between two taxonomic groups. In addition, the variance of each taxonomic group is indicated in the gray legend box.

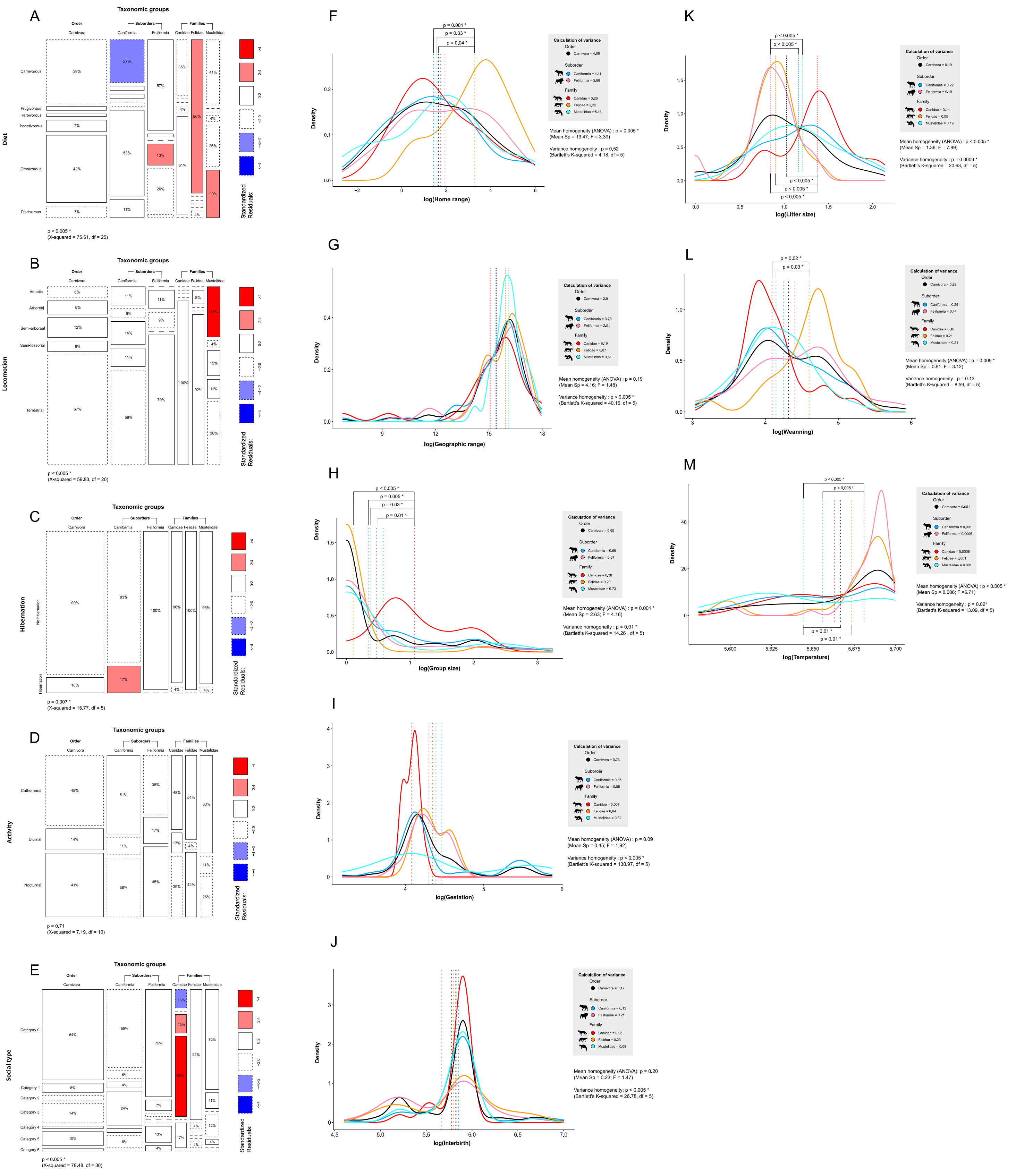

Supplementary Table 1. Percentage of correct identification of significant shifts in evolutionary rates of the relative brain size identified in the random computed topologies.

Node	% Accelerated rate of evolution	% Deccelerated rate of evolution
Herpestidae + Hyaenidae + Eupleridae	1%	23%
Canidae	81%	0%
Helictindinae + Guloninae + Ictonychinae + Mustelinae + Lutrinae (Mustelidae)	76%	0%

Supplementary Table 2. Species values of each variable related to ecology, environment, social complexity, and life-trait history used in PGLS analyses (dataset 2, 124 species).

Diet: Carn: carnivorous; Fru: frugivorous; Herb: herbivorous and folivorous; Inv: invertivorous (i.e. invertebreate-eaters); Omn: omnivorous; Pisc: piscivorous;

Locomotion: Aq: aquatic; Arb: arboreal; Sarb: semi-arboreal; Sfos: semi-fossorial; Terr: terrestrial.

Activity pattern: Cath/Cre: Cathemeral or crepuscular; Diu: Diurnal; Noc: Nocturnal.

Species	Family	Average body mass (g)	Diet	Locomotion	Hibernation	Activity pattern	Sociale complexity	Group size	Gestation (days)	Interbirth (days)	Weaning (days)	Geographic range (km^2)	Home range (km^2)	Mean temperature (K)
Acinonyx jubatus	Felidae	50577.92	Carn	Terr	No	Diu	1	1.4	92	547.5	91.42	10586306.53	88.27	296.233
Ailuropoda melanoleuca	Ursidae	117999.99	Herb	Terr	No	Cath/Cre	0	1	127.5	638.75	178.98	36206.89	3.55	279.7
Ailurus fulgens	Ailuridae	5170.08	Herb	Arb	No	Cath/Cre	0	1	135	365	136.87	1075406.59	1.43	277.623
Amblonyx cinereus	Mustelidae	3527.59	Pisc	Aq	No	Noc	4	12.5	63	182.5	79.54	3673360.97	17	295.807
Aonyx capensis	Mustelidae	19322.22	Pisc	Aq	No	Cath/Cre	1	1	62.99	365	52.5	14162350.57	99.74	296.46
Arctictis binturong	Viverridae	12999.99	Omn	Arb	No	Noc	0	1	91.5	317.6	117.57	2913838.88	6.2	296.144
Arctogalidia trivirgata	Viverridae	2323.79	Omn	Arb	No	Noc	0	1	45	182.5	49.41	2766097.4	11.34	296.36
Atilax paludinosus	Herpestidae	3600.16	Omn	Terr	No	Cath/Cre	0	1	74.5	182.5	35.89	14296320.45	1.73	296.489
Bassaricyon alleni	Procyonidae	1235.01	Fru	Arb	No	Noc	0	1	74	182.5	35.89	441326.32	0.375	297.139
Bassariscus astutus	Procyonidae	1010.37	Omn	Sarb	No	Noc	0	1	52.5	365	52.89	3916072.44	0.74	288.549
Bassariscus sumichrasti	Procyonidae	906	Omn	Sarb	No	Noc	0	1	64	365	119.32	625977.74	0.2	296.181
Canis aureus	Canidae	9658.7	Omn	Terr	No	Cath/Cre	3	2.75	63	365	61.3	25739527.43	2.95	294.873
Canis latrans	Canidae	11989.1	Omn	Terr	No	Cath/Cre	3	4.33	63	365	43.71	17099094.3	18.88	278.968
Canis lupus	Canidae	31756.51	Carn	Terr	No	Cath/Cre	5	7	63	365	44.82	50803439.7	159.86	273.632
Canis simensis	Canidae	14361.86	Carn	Terr	No	Diu	5	8	61	365	69.6	11402.81	4.2	283.053
Caracal caracal	Felidae	11964.38	Carn	Terr	No	Noc	0	1	74.5	365	120.41	26609096.78	293.94	295.307
Catopuma temminckii	Felidae	7726.46	Carn	Terr	No	Noc	0	1	79	365	180	3437288.13	40.15	292.874
Cerdocyon thous	Canidae	5741.66	Omn	Terr	No	Noc	3	2	56	243.33	72.33	7091683.41	1.41	296.146
Chrysocyon brachyurus	Canidae	23325	Omn	Terr	No	Cath/Cre	0	2	63.48	365	119	5038801	21.36	295.499
Civettictis civetta	Viverridae	12075.58	Omn	Terr	No	Noc	0	1	70.5	212.14	82.91	14207678.6	11.1	296.864
Conepatus chinga	Mephitidae	1918	Omn	Sfos	Yes	Noc	0	1	60	365	63	3247304.52	16.67	286.541
Conepatus leuconotus	Mephitidae	3293.91	Omn	Sfos	Yes	Noc	0	1	70	182.5	45.56	2509559.21	1.27	291.222
Crocuta crocuta	Hyaenidae	63369.98	Carn	Terr	No	Noc	6	25.5	112.3	441.04	371.37	14790149.69	21.93	296.996
Cryptoprocta ferox	Eupleridae	9500	Carn	Arb	No	Cath/Cre	0	1	92	365	136.1	214312.12	0.75	295.348
Cuon alpinus	Canidae	15800	Omn	Terr	No	Cath/Cre	5	7.5	63	365	57.33	10546280.1	33.64	282.798
Cynictis penicillata	Herpestidae	694.41	Inv	Terr	No	Diu	5	3.5	56	365	41.76	2233627.48	1.89	291.57
Eira barbara	Mustelidae	4134.99	Omn	Arb	No	Cath/Cre	0	1	65	365	94.46	13411787.9	10.7	296.732
Felis chaus	Felidae	7157.99	Carn	Terr	No	Cath/Cre	0	1	64.5	130.01	95.49	8986835.66	112.5	293.257
Felis margarita	Felidae	2823.36	Carn	Terr	No	Noc	0	1	63	197.7	120	12141993.7	9	295.537
Felis silvestris	Felidae	4573.08	Carn	Terr	No	Cath/Cre	0	1	60.5	148.25	76.01	32110980.01	1.29	292.428
Fossa fossana	Eupleridae	1853.98	Omn	Terr	No	Noc	0	1	84.5	365	66.84	106522.05	0.75	293.431
Galerella sanguinea	Herpestidae	543.83	Inv	Terr	No	Diu	1	1	65	182.5	54.44	16368169.48	0.66	296.477
Galidia elegans	Eupleridae	810	Omn	Sarb	No	Cath/Cre	0	1	75	365	59.27	120561.29	0.17	294.35
Galidictis fasciata	Eupleridae	974.995	Carn	Terr	No	Cath/Cre	0	1	85.5	365	60	87237.59	1.08	292.928
Genetta genetta	Viverridae	1756.17	Carn	Sarb	No	Noc	0	1	73.5	182.5	71.8	17766920.65	11.54	296.049
Genetta maculata	Viverridae	1950	Carn	Sarb	No	Noc	0	1	73.5	182.5	42	12805950.62	4.35	296.469
Gulo gulo	Mustelidae	12792.49	Carn	Terr	No	Noc	0	1	242.5	821.25	83.64	24495951.71	343.27	265.945
Helarctos malayanus	Ursidae	57075.78	Omn	Terr	No	Noc	0	1	105	100	90.36	2525372.13	12	297.538
Helogale parvula	Herpestidae	281.83	Inv	Terr	No	Diu	6	11,0	49	141.94	21	6944753.53	0.21	295.394
Herpailurus yagouaroundi	Felidae	6875	Carn	Terr	No	Cath/Cre	0	1	72.5	182.5	48.46	15093388.56	52.35	296.503
Herpestes ichneumon	Herpestidae	2980.02	Omn	Terr	No	Cath/Cre	1	1	60	365	58.09	14429050.1	2.18	296.364
Hyaena hyaena	Hyaenidae	35070.51	Carn	Terr	No	Cath/Cre	1	2.3	90.5	237.25	118.93	24254343.2	45.19	296.047
Hydrictis maculicollis	Mustelidae	4180.53	Pisc	Aq	No	Cath/Cre	0	1	59.6	365	60.48	11363947.46	11	296.921
Ichneumia albicauda	Herpestidae	3628.4	Inv	Terr	No	Noc	0	1	60	365	270	14326821.09	0.5	296.866
Leopardus geoffroyi	Felidae	3590	Carn	Terr	No	Noc	0	1	72	240	75	3653843.2	274.75	288.873
Leopardus pardalis	Felidae	11880	Carn	Terr	No	Cath/Cre	0	1	80.5	360.9	106.18	16040024.42	5.38	296.019
Leptailurus serval	Felidae	11999.96	Carn	Terr	No	Cath/Cre	0	1	74	182.5	104.68	12526196.74	1.84	296.707
Lontra canadensis	Mustelidae	8087.42	Pisc	Aq	No	Cath/Cre	3	15	62	365	121.66	11887021.15	16.95	274.213
Lontra longicaudis	Mustelidae	6554.97	Pisc	Aq	No	Cath/Cre	0	1	71	365	180	12885820.36	5.5	296.656
Lupulella adusta	Canidae	10392.49	Omn	Terr	No	Noc	3	2	60	365	52.89	10581413.14	1.01	296.801
Lupulella mesomelas	Canidae	8247.3	Omn	Terr	No	Cath/Cre	3	2	60	273.75	34.1	6248779.95	11.93	294.483
Lutra lutra	Mustelidae	8868.69	Pisc	Aq	No	Noc	1	1	61.5	365	100.8	25877469.45	25.48	278.176
Lutrogale perspicillata	Mustelidae	8912.49	Pisc	Aq	No	Diu	3	8	61.5	365	72.53	5506788.05	5.31	297.858
Lycalopex culpaeus	Canidae	8616.24	Carn	Terr	No	Noc	0	1	58	365	48.22	2809619.42	5.35	282.025
Lycalopex griseus	Canidae	3000	Omn	Terr	No	Cath/Cre	3	2.5	55.5	365	180	1620863.23	2	284.289
Lycaon pictus	Canidae	21999.99	Carn	Terr	No	Diu	5	8	72	355.87	27.28	10539675.81	395.88	296.705
Lynx canadensis	Felidae	9682.82	Carn	Terr	No	Noc	0	1	63.4	365	111.36	8252985.31	30.6	269.974
Lynx lynx	Felidae	19300	Carn	Terr	No	Cath/Cre	0	1	70	365	81.85	23827627.14	136.35	271.426
Lynx rufus	Felidae	6374.47	Carn	Terr	No	Cath/Cre	0	1	62	365	59.99	9702533.02	31.2	283.981
Martes americana	Mustelidae	873.69	Carn	Sarb	No	Cath/Cre	0	1	247.5	365	45.73	7620172.14	3.66	270.538
Martes flavigula	Mustelidae	2504.64	Omn	Sarb	No	Diu	3	2	255	182.5	89.49	5260689.7	15	288.059
Martes foina	Mustelidae	1675	Omn	Sarb	No	Cath/Cre	0	1	252.5	365	74.11	7011029.37	2.15	280.028
Martes martes	Mustelidae	1299.99	Omn	Sarb	No	Noc	0	1	252.5	365	54.19	8361437.29	2.15	277.405
Martes pennanti	Mustelidae	3750	Carn	Terr	No	Cath/Cre	0	1	355	365.01	84	3481123.82	15.96	274.075
Martes zibellina	Mustelidae	1173.91	Carn	Sarb	No	Cath/Cre	0	1	275	365	52.23	6492749.47	15.2	265.322
Mellivora capensis	Mustelidae	8999.99	Omn	Sfos	No	Cath/Cre	1	1	60	182.5	23.86	27823522.11	333.5	296.806
Melogale moschata	Mustelidae	938.5	Inv	Sfos	No	Cath/Cre	0	1	70	365	75	1476533.73	0.65	289.526
Melursus ursinus	Ursidae	99999.99	Omn	Terr	Yes	Cath/Cre	0	1	203.4	1095	194.67	417387.69	8.15	297.329
Mephitis mephitis	Mephitidae	2399.99	Omn	Sfos	Yes	Noc	0	1	68	365	55.15	12277338.73	2.32	280.747
Mungos mungo	Herpestidae	1260	Inv	Terr	No	Diu	5	23.7	63	365	20.88	15894555.38	1.66	297.005
Mungotictis decemlineata	Eupleridae	657.03	Omn	Terr	No	Diu	5	4	97.5	365	45.1	17099.69	1.19	297.282
Mustela erminea	Mustelidae	284.5	Carn	Terr	No	Cath/Cre	0	1	66.4	365	60.93	39718064.94	0.11	270.328
Mustela frenata	Mustelidae	190.03	Carn	Terr	No	Cath/Cre	0	1	24.5	365	34.23	12290918.8	0.21	286.243
Mustela lutreola	Mustelidae	566.44	Carn	Terr	No	Cath/Cre	0	1	53.5	365	58.71	3568955.39	0.17	277.331
Mustela nivalis	Mustelidae	78.45	Carn	Terr	No	Cath/Cre	0	1	35.5	273.75	36.74	43656881.02	0.07	272.442
Mustela putorius	Mustelidae	975.55	Carn	Terr	No	Noc	0	1	42	252.5	54.39	7565541.29	0.53	279.538
Mustela sibirica	Mustelidae	530.85	Carn	Terr	No	Cath/Cre	0	1	35	365	58.2	20179461.96	3.41	275.398
Nandinia binotata	Nandiniidae	2167.2	Omn	Arb	No	Noc	0	1	64	182.5	63.81	5977079.52	0.55	297.147
Nasua narica	Procyonidae	4578.43	Omn	Sarb	No	Cath/Cre	5	6.5	73.5	365	132.63	2329129.95	0.78	293.711
Nasua nasua	Procyonidae	3775.5	Omn	Sarb	No	Diu	5	20	75.5	365	113.15	10694990.22	0.79	297.066
Neofelis nebulosa	Felidae	14945.05	Carn	Arb	No	Cath/Cre	0	1	91.5	390	111.67	2913590.85	35	294.979
Neovison vison	Mustelidae	945	Carn	Terr	No	Noc	0	1	57.5	365	42	13218592.49	4.35	274.641
Nyctereutes procyonoides	Canidae	4214.99	Omn	Terr	Yes	Noc	2	2	63	365	49.69	6704451.99	1.15	278.823
Otocyon megalotis	Canidae	4098.12	Inv	Terr	No	Cath/Cre	3	4.3	67.5	182.5	42.46	5001822.67	1.13	294.158
Paguma larvata	Viverridae	4300	Omn	Sarb	No	Noc	0	1	53.5	182.5	109.13	5422715.54	2.88	290.761
Panthera leo	Felidae	158623.93	Carn	Terr	No	Noc	5	8.7	110	730	197.86	11237685.34	55.19	296.588
Panthera onca	Felidae	83943.09	Carn	Terr	No	Cath/Cre	0	1	99	365	156.6	10500825.25	52.55	297.353
Panthera pardus	Felidae	52399.99	Carn	Terr	No	Cath/Cre	0	1	96	476.37	123.54	29348601.36	18.78	293.913
Panthera tigris	Felidae	161914.66	Carn	Terr	No	Noc	0	1	103	821.25	118.55	2494993.45	64.89	294.301
Panthera uncia	Felidae	32500	Carn	Terr	No	Cath/Cre	0	1	99	365	101.42	2744453.48	24.07	268.469
Paradoxurus hermaphroditus	Viverridae	3200	Fru	Sarb	No	Noc	0	1	62	182.5	60	7074837.93	4.15	296.409
Parahyaena brunnea	Hyaenidae	42977.93	Carn	Terr	No	Noc	5	4.8	96	532.29	302.44	1981470.71	67.3	291.81
Pardofelis marmorata	Felidae	2826.68	Carn	Arb	No	Noc	0	1	74	365	59	2298300.89	5.3	295.839
Potos flavus	Procyonidae	2441.81	Fru	Arb	No	Noc	4	4	110	365	109.13	12770952.51	0.22	297.299
Prionailurus bengalensis	Felidae	2780.97	Carn	Terr	No	Noc	0	1	65	165.56	24.85	10201747.8	1.87	290.06
Prionailurus planiceps	Felidae	3533.76	Pisc	Terr	No	Noc	0	1	56	365	180	1100389.34	13	298.18
Prionailurus viverrinus	Felidae	8826.46	Carn	Terr	No	Cath/Cre	0	1	66.5	365	135.96	3546617.17	13	296.546
Procyon cancrivorus	Procyonidae	6931.72	Omn	Terr	No	Noc	0	1	63	365	111.36	12829315.4	6.1	296.893
Procyon lotor	Procyonidae	6373.72	Omn	Sarb	Yes	Cath/Cre	1	1	63	365	104.16	11703644.72	4.9	284.334
Proteles cristata	Hyaenidae	8139.39	Inv	Terr	No	Noc	2	1.25	90	365	120.48	6435462.88	1.51	294.158
Pseudalopex gymnocercus	Canidae	4542.67	Carn	Terr	No	Noc	0	1	59.23	365	90	2321073.61	0.42	291.683
Pteronura brasiliensis	Mustelidae	26000	Pisc	Aq	No	Diu	3	8	67.5	365	125.73	8075624.21	85.849	297.694
Puma concolor	Felidae	53954.05	Carn	Terr	No	Cath/Cre	0	1	94	577.91	120.48	22350859.07	129.89	291.343
Speothos venaticus	Canidae	6324.54	Carn	Terr	No	Diu	3	7	67	247.94	127.3	9703966.13	4.65	297.197
Spilogale gracilis	Mephitidae	466.5	Omn	Sfos	Yes	Noc	0	1	220	365	60	3831634.7	0.455	284.915
Spilogale putorius	Mephitidae	566.49	Omn	Sfos	Yes	Noc	0	1	60	243.33	55.68	2957350.08	0.29	286.566
Suricata suricatta	Herpestidae	729.99	Inv	Terr	No	Diu	5	6.35	70	365	55.68	1720747.74	14.06	290.731
Taxidea taxus	Mustelidae	7842.15	Omn	Sfos	Yes	Noc	0	1	43.8	365	41.99	8860573.16	3.44	282.304
Tremarctos ornatus	Ursidae	123176.97	Omn	Terr	No	Cath/Cre	0	1	210	365	173.44	409517.73	17	291.132
Urocyon littoralis	Canidae	1923	Omn	Terr	No	Cath/Cre	3	2	53.7	365	63	882.7	0.45	298.75
Urocyon cinereoargenteus	Canidae	3833.71	Omn	Terr	No	Cath/Cre	2	2	60	365	63.79	9010481.09	1.83	287.991
Ursus americanus	Ursidae	110500	Omn	Terr	Yes	Diu	0	1	214	821.25	167.99	9650153.48	31.25	273.539
Ursus arctos	Ursidae	196287.5	Omn	Terr	Yes	Cath/Cre	0	1	227.5	912.5	182.5	23972321.42	333.82	268.494
Ursus thibetanus	Ursidae	99714.19	Omn	Terr	Yes	Cath/Cre	0	1	210	365	82.01	5348705.49	0.7	285.814
Urva edwardsii	Herpestidae	1304.67	Omn	Terr	No	Diu	0	1	62	109.5	34.8	3629768.58	0.155	297.384
Urva javanica	Herpestidae	750	Omn	Terr	No	Diu	0	1	48.5	152.08	43.59	6880297.87	0.22	293.968
Viverra zibetha	Viverridae	9148.77	Omn	Terr	No	Noc	0	1	80.3	182.5	30	4064204.47	9.359	291.94
Viverricula indica	Viverridae	2918.88	Omn	Terr	No	Cath/Cre	0	1	67	365	127.5	8147745.62	2.7	294.819
Vormela peregusna	Mustelidae	594.13	Omn	Terr	No	Cath/Cre	0	1	285	365	30	8883616.47	0.55	280.183
Vulpes chama	Canidae	2919.80	Carn	Terr	No	Noc	2	2	52.59	365	70	2079866.13	2.8	291.06
Vulpes lagopus	Canidae	3584.37	Carn	Terr	No	Cath/Cre	3	2	52.5	365	49.5	1774767	65	273.47
Vulpes rueppellii	Canidae	3249.97	Omn	Terr	No	Noc	3	3.8	52.5	365	49	11669070.9	29.93	295.333
Vulpes velox	Canidae	2088	Omn	Terr	No	Noc	3	2.5	51	365	47.08	671658.66	4.59	283.712
Vulpes vulpes	Canidae	4820.36	Omn	Terr	No	Noc	3	3	52	365	50.71	63034304.34	3.5	276.582

Supplementary Table 3. PGLS analyses performed on each 13 variables separately using the entire carnivoran dataset (N= 124). Asterisks indicate the level of significance of p-values (*: < 0.05; **: < 0.01; and ***: < 0.001 respectively).

Diet			
Predictor	SE	*F*	*p*
Diet	0.002	1.41	0.23
Model summary: λ = 0.67, R^2 = 0.02, p = 0.23			

Locomotion			
Predictor	SE	*F*	*p*
Locomotion	0.001	0.76	0.55
Model summary: λ = 0.70, R^2 = -0.007, p = 0.55			
Activity pattern			
Predictor	SE	*F*	*p*
Activity pattern	0.001	0.35	0.71
Model summary: λ = 0.70, R^2 = -0.01, p = 0.71			
Home range			
Predictor	SE	*F*	*p*
Home range	0.006	4.03	0.046*
Model summary: λ = 0.61, R^2 = 0.02, p = 0.046*			
Geographic range			
Predictor	SE	*F*	*p*
Geographic range	0.03	14.87	< 0.001***
Model summary: λ = 0.83, R^2 = 0.10, p = < 0.001***			
Mean temperature			
Predictor	SE	*F*	*p*
Mean temperature	0.0005	0.27	0.6
Model summary: λ = 0.71, R^2 = -0.006, p = 0.6			

Ability to hibernate			
Predictor	SE	*F*	*p*
Hibernation	0.006	3.44	0.07
Model summary: λ = 0.69, R^2 = 0.02, p = 0.07			
Gestation length			
Predictor	SE	*F*	*p*
Gestation length	0.002	0.83	0.36
Model summary: λ = 0.73, R^2 = -0.001, p = 0.36			
Weaning time			
Predictor	SE	*F*	*p*
Weaning time	0.006	3.75	0.055
Model summary: λ = 0.67, R^2 = 0.02, p = 0.055			

Interbirth length			
Predictor	SE	*F*	*p*
Interbirth length	0.001	0.45	0.50
Model summary: λ = 0.69, R^2 = -0.004, p = 0.50			

Litter size			
Predictor	SE	*F*	*p*
Litter size	0.007	4.35	0.04*
Model summary: λ = 0.67, R^2 = 0.03, p = 0.04*			

Group size			
Predictor	SE	*F*	*p*
Group size	0.001	0.78	0.40
Model summary: λ = 0.69, R^2 = -0.002, p = 0.40			

Social complexity			
Predictor	SE	*F*	*p*
Social complexity	0.01	0.97	0.45
Model summary: λ = 0.72, R^2 = -0.001, p = 0.45			
