# Supplementary material for: The impact of environmental factors on the evolution of brain size in carnivorans
Source: Commun Biol. 2022 Sep 21;5:998. doi: 10.1038/s42003-022-03748-4 (PMC9492690; doi:10.1038/s42003-022-03748-4)
Supplement: Supplementary file 3 — Description of Additional Supplementary Files [file 42003_2022_3748_MOESM3_ESM.pdf]

Description of Additional Supplementary Files

File name: Supplementary Code 1
Description: The RCode used in this study.

File name: Supplementary Data 1
Description: List of specimens studied and associated raw external cranial measurements.

File name: Supplementary Data 2
Description: List of species, number of specimens (N) and associated estimated brain volume, average body mass and phylogenetic encephalization quotient used in this study. Brain volumes extracted from Finarelli (2006) are indicated with an asterisk.
