# Supplementary material for: The impact of environmental factors on the evolution of brain size in carnivorans
Source: Commun Biol. 2022 Sep 21;5:998. doi: 10.1038/s42003-022-03748-4 (PMC9492690; doi:10.1038/s42003-022-03748-4)
Supplement: Supplementary file 4 — Supplementary Data 1 and 2 [file 42003_2022_3748_MOESM4_ESM.pdf]

Supplementary Data 1. List of specimens studied and associated raw external cranial measurements.
Measurements: Width (W), length (L) and height (H) used to estimates the brain volume.
Collection acronyms: AMNH: American Museum of Natural History (New York, NY, USA); FMNH: The Field Museum of Natural History (Chicago,IL, USA); LACM: Natural History Museum of Los Angeles County (Los Angeles, CA, USA); MNHN: Museum National d'Histoire Naturel (Paris, France); MVZ: Museum of Vertebrate Zoology (Berkeley, CA, USA); NHMUK: Natural History Museum (former British Museum of Natural History, London, UK); RMCA: Royal Museum for Central Africa (Tervuren, Belgium); USNM: Smithsonian National Museum of Natural History (United States National Museum; Washington, DC, USA);
Sex: F: Female; M: Male; U: Unknown;
Origin: C: Raise in captivity; W: Wild caught; U: Unknown.

Species	Collection ID	Online collection ID	Family	W	L	H	Measured brain volume (ml)	Sexe	Origin
Acinonyx jubatus	MNHN-ZM-AC 1941-51		Felidae	71.27	117.64	57.89	124.64	U	U
Acinonyx jubatus	RMCA 83001M-28		Felidae	68.68	123.09	52.76	109.15	M	C
Ailuropoda melanoleuca	Macho MAV 3156	Phenome10k<br>(provided by Borja Figueirido)	Ursidae	94.75	195.77	80.57	292.98	U	U
Ailurus fulgens	MNHN-ZM-MO 1960-84		Ailuridae	72.54	77.24	26.53	49.55	F	C
Ailurus fulgens	MNHN-ZM-MO 1960-85		Ailuridae	79.20	80.59	25.05	52.75	M	C
Ailurus fulgens	MNHN-ZM-MO 1963-358		Ailuridae	46.72	90.26	27.39	30.62	F	C
Amblonyx cinereus	MNHN-ZM-MO 1982-165		Mustelidae	47.59	75.13	29.49	32.20	M	W
Amblonyx cinereus	MNHN-ZM 2005-607		Mustelidae	43.90	63.76	29.55	27.82	U	U
Arctictis binturong	MNHN-ZM-MO 1936-1529		Viverridae	49.58	84.42	30.76	36.65	U	C
Arctictis binturong	MNHN-ZM-MO 1990-88		Viverridae	49.26	100.99	34.52	43.19	M	C
Arctogalidia trivirgata	MNHN-ZM-MO 1998-1970		Viverridae	33.11	83.12	24.47	17.39	M	C
Arctogalidia trivirgata	MNHN-ZM-MO 2001-495		Viverridae	33.25	89.07	24.34	17.48	M	C
Arctonyx collaris	MNHN-ZM-AC 1961-186		Mustelidae	48.87	102.43	45.16	57.07	M	U
Arctonyx collaris	MNHN-ZM-MO 1962-1638		Mustelidae	52.95	101.41	43.70	60.94	U	W
Atilax paludinosus	MNHN-ZM-MO 1933-2856		Herpestidae	36.94	73.73	27.81	21.82	U	C
Atilax paludinosus	MNHN-ZM-MO 1995-2679		Herpestidae	35.56	75.72	30.03	24.58	U	U
Atilax paludinosus	MNHN-ZM-MO 1995-427		Herpestidae	36.52	86.88	30.21	24.58	M	W
Bassaricyon alleni	MCZ 37920		Procyonidae	35.59	60.58	18.82	13.02	U	U
Bassaricyon alleni	MCZ 37922		Procyonidae	34.31	60.03	17.72	11.63	F	U
Bassaricyon alleni	MCZ 37923		Procyonidae	34.67	59.84	18.01	11.98	M	U
Bassaricyon gabbii	MNHN-ZM-MO 1983-945		Procyonidae	35.21	60.97	18.42	12.58	U	W
Bassariscus astutus	MNHN-ZM-MO 1898-1483		Procyonidae	32.20	57.90	19.10	11.50	U	W
Bassariscus astutus	MNHN-ZM-MO 1911-768		Procyonidae	33.08	57.43	19.73	12.29	U	U
Bassariscus astutus	MNHN-ZM-MO A1619		Procyonidae	32.75	60.22	19.87	12.39	U	U
Bdeogale crassicauda	MNHN-ZM-MO 1881-1521		Herpestidae	34.65	69.51	24.03	16.95	M	W
Bdeogale crassicauda	MNHN-ZM-MO 1921-29		Herpestidae	30.49	65.35	21.78	12.76	U	U
Bdeogale nigripes	RMCA 92-124-M-001		Herpestidae	40.25	81.10	29.16	26.28	M	W
Canis aureus	MNHN-ZM-AC 1923-616		Canidae	52.39	89.67	40.58	53.62	M	W
Canis aureus	MNHN-ZM-MO 1972-384		Canidae	49.43	87.70	38.24	46.48	F	W
Canis latrans	MNHN-ZM 2007-454		Canidae	56.57	109.31	46.56	72.29	U	W
Canis latrans	MNHN-ZM-MO 1962-967		Canidae	58.41	108.12	45.83	73.81	U	U
Canis lupus	LACM 23010	Morphosource Media ID<br>000009407<br>(provided by Jack Tseng)	Canidae	75.33	153.39	59.58	148.50	U	U
Canis lupus	MNHN-ZM-AC 1941-284		Canidae	58.59	100.10	61.95	99.81	U	U
Canis lupus	MNHN-ZO-AC 1942-130		Canidae	65.78	118.11	57.58	112.06	M	C
Canis lupus	MNHN-ZM 2015-1255		Canidae	72.47	131.04	58.39	132.42	U	U
Canis simensis	MNHN-ZM-MO 1962-1508		Canidae	58.97	109.79	49.12	80.75	U	U
Canis simensis	MNHN-ZM-MO 1962-1509		Canidae	62.35	119.55	51.67	93.66	U	U
Caracal caracal	MNHN-ZM-MO 1919-25		Felidae	53.56	88.55	31.88	42.55	U	C
Caracal caracal	MNHN-ZM-MO 1969-455		Felidae	57.83	98.21	36.86	56.32	M	W
Caracal caracal	MNHN-ZM 2006-530		Felidae	56.57	88.93	34.41	49.53	M	W
Catopuma temminckii	MNHN-ZM-MO 1939-2152		Felidae	65.57	103.16	42.34	77.57	F	U
Catopuma temminckii	MNHN-ZM-AC 1941-293		Felidae	57.27	108.85	40.26	62.86	U	C
Cerdocyon thous	MNHN-ZM-MO 1962-1562		Canidae	49.15	80.14	33.09	38.60	U	W
Chrotogale owstoni	NHMUK 33.4.1.229		Viverridae	36.16	72.90	27.05	20.56	U	U
Civettictis civetta	MNHN-ZM-MO 1969-462		Viverridae	43.84	96.29	32.54	34.52	F	C
Civettictis civetta	MNHN-ZM-MO 1971-89		Viverridae	42.27	98.06	33.37	34.02	U	U
Civettictis civetta	RMCA 21-554		Viverridae	44.48	89.72	34.05	36.17	F	W
Conepatus chinga	MNHN-ZM-AC 1897-432		Mephitidae	31.56	45.61	22.03	12.20	U	U
Conepatus chinga	MNHN-ZM 2005-649		Mephitidae	29.06	44.41	21.05	10.39	F	W
Conepatus leuconotus	MNHN-ZM-MO 1868-1045		Mephitidae	34.40	53.92	28.19	18.53	U	W
Crocuta crocuta	LACM 30655	Phenome10k<br>(provided by Jack Tseng)	Hyaenidae	75.65	154.21	62.59	157.54	U	U
Crocuta crocuta	MNHN-ZM-AC 1936-656		Hyaenidae	77.01	179.94	65.66	177.03	U	C
Crocuta crocuta	MNHN-ZM-MO 1972-399		Hyaenidae	91.75	169.37	64.36	212.84	U	W
Crocuta crocuta	NA	African fossils (https://africanfossi	Hyaenidae	78.26	179.52	70.37	194.32	U	U
Crossarchus obscurus	MNHN-ZM-MO 1961-1027		Herpestidae	26.89	52.56	20.51	9.60	M	C
Crossarchus obscurus	MNHN-ZM-MO 1976-333		Herpestidae	27.11	49.50	19.23	8.91	U	W
Crossarchus obscurus	MNHN-ZM-MO 2000-1062		Herpestidae	27.05	47.51	18.21	8.29	U	U
Crossarchus obscurus	MNHN-ZM-MO 2000-1067		Herpestidae	26.48	51.71	21.09	9.66	U	U
Cryptoprocta ferox	MNHN-ZM-AC 1927-227		Eupleridae	42.41	89.69	31.95	31.82	U	W
Cryptoprocta ferox	MNHN-ZM-MO 1940-1211		Eupleridae	45.60	94.33	35.74	39.85	M	C
Cryptoprocta ferox	MNHN-ZO-MO 1977-554		Eupleridae	43.84	95.82	35.44	37.74	M	W
Cryptoprocta ferox	MNHN-ZM-MO 1992-1667		Eupleridae	42.57	85.22	31.22	30.76	U	W
Cryptoprocta ferox	MNHN-ZM-MO 1992-1668		Eupleridae	46.22	87.22	33.42	36.94	U	W
Cryptoprocta ferox	MNHN-ZM-AC A12808		Eupleridae	45.43	90.24	34.19	37.38	U	U
Cuon alpinus	MNHN-ZM-MO 1927-1252		Canidae	62.65	113.02	52.25	93.86	F	W
Cuon alpinus	MNHN-ZM 2007-473		Canidae	63.21	108.13	49.73	88.97	U	W
Eira barbara	MNHN-ZM-MO 2000-646		Mustelidae	44.46	92.15	34.94	37.43	M	U

Enhydra lutris	MNHN-ZM-AC 1935-124		Mustelidae	73.54	97.72	51.67	109.16	U	W
Enhydra lutris	MNHN-ZM-MO 1962-2678		Mustelidae	76.27	105.33	55.80	126.67	U	U
Eupleres goudotii	MNHN-ZM-MO 1962-1602		Eupleridae	33.37	56.10	23.64	14.95	U	W
Eupleres goudotii	MNHN-ZM-MO 1962-2091		Eupleridae	33.33	60.46	22.98	14.80	U	U
Eupleres goudotii	MNHN-ZM-MO 1962-2105		Eupleridae	31.96	59.83	23.60	14.39	M	W
Eupleres goudotii	NHMUK 35.1.8.308		Eupleridae	32.08	59.63	21.88	13.33	M	U
Felis chaus	MNHN-ZM-MO 1845-120		Felidae	46.47	82.23	30.94	33.71	U	W
Felis margarita	MNHN-ZM-MO 1962-2933		Felidae	43.30	64.88	23.53	21.58	U	U
Felis margarita	MNHN-ZM 2014-566		Felidae	44.11	65.60	23.22	21.85	M	W
Felis silvestris	MNHN CaE492		Felidae	40.15	66.96	24.49	20.63	U	U
Felis silvestris	MNHN-ZM-AC 1880-498		Felidae	46.68	87.29	27.55	30.49	U	U
Felis silvestris	MNHN-ZM 2006-525		Felidae	44.85	75.40	25.11	25.21	M	U
Fossa fossana	MNHN-ZM-MO 1932-3556		Eupleridae	31.92	71.18	23.56	15.05	M	W
Fossa fossana	MNHN-ZM-MO 1932-3557		Eupleridae	34.07	66.88	23.80	16.24	M	W
Fossa fossana	MNHN-ZM-MO 1962-1597		Eupleridae	34.00	66.26	24.33	16.54	U	W
Fossa fossana	MNHN-ZM-MO 1962-1598		Eupleridae	32.68	64.07	22.71	14.49	M	W
Fossa fossana	MNHN-ZM-MO 1962-2112		Eupleridae	34.27	66.41	22.48	15.38	U	W
Fossa fossana	MNHN-ZM 2006-568		Eupleridae	31.56	68.71	23.20	14.46	U	U
Galerella sanguinea	MNHN-ZM-MO 1918-17		Herpestidae	24.04	43.30	15.55	5.88	U	W
Galerella sanguinea	MNHN-ZM-MO 2001-2188		Herpestidae	26.95	55.22	17.68	8.34	M	W
Galerella sanguinea	USNM 539732	Digimorph (provided by Blaire Van Valkenburgh & Tim Rowe)	Herpestidae	25.22	51.80	17.83	7.60	M	W
Galictis cuja	MNHN-ZM_MO 1960-3811		Mustelidae	34.73	66.02	26.29	18.43	U	C
Galictis cuja	MNHN-ZM 2005-651		Mustelidae	31.98	60.25	25.53	15.68	U	W
Galictis vittata	MNHN-ZM-MO 2001-1971		Mustelidae	40.27	71.78	30.57	26.72	M	W
Galidia elegans	MNHN-ZM-MO 1998-1938		Eupleridae	29.90	57.24	19.68	10.77	F	W
Galidia elegans	MNHN-ZM-MO 1912-124		Eupleridae	28.27	54.69	20	10.07	U	W
Galidia elegans	MNHN-ZM-MO 1932-3529		Eupleridae	29.53	52.57	19.86	10.45	M	W
Galidia elegans	MNHN-ZM-MO 1932-3534		Eupleridae	27.42	49.48	17.80	8.33	F	W
Galidia elegans	MNHN-ZM-AC 1951-2		Eupleridae	26.92	50.15	18.96	8.73	U	W
Galidia elegans	MNHN-ZM-MO 1962-2066		Eupleridae	26.15	50.14	17.91	7.92	U	W
Galidia elegans	MNHN-ZM-MO 1962-2070		Eupleridae	27.41	54.45	19.12	9.22	U	W
Galidia elegans	MNHN-ZM-MO 1962-2071		Eupleridae	29.82	53.93	19.26	10.32	M	W
Galidia elegans	MNHN-ZM-MO 1962-2109		Eupleridae	29.67	57.22	20.13	10.92	M	W
Galidia elegans	MNHN-ZM-MO 1974-316		Eupleridae	27.40	54.56	18.25	8.78	F	C
Galidia elegans	MNHN-ZM-MO 1992-1677		Eupleridae	28.22	49.41	18.39	8.94	F	C
Galidictis fasciata	MNHN-ZM-MO 1932-3539		Eupleridae	27.76	53.58	19.45	9.50	M	W
Galidictis fasciata	NHMUK 1938.11.16.2		Eupleridae	27.71	51.25	20.19	9.74	U	U
Galidictis fasciata	NHMUK 1938.11.16.3		Eupleridae	29	48.35	18.93	9.48	U	U
Galidictis fasciata	NHMUK 1938.11.16.4		Eupleridae	29.65	50.49	19.42	10.15	U	U
Genetta bourloni	MNHN-ZM-MO 2003-427		Viverridae	30.50	66.82	21.62	12.74	U	W
Genetta bourloni	MNHN-ZM-MO 2003-435		Viverridae	33.30	69.05	22.23	14.81	U	W
Genetta genetta	MNHN-ZM-MO 1962-2065		Viverridae	29.38	65.65	19.56	10.88	U	W
Genetta genetta	MNHN-ZM-AC 1963-368		Viverridae	29.60	63.82	19.26	10.72	F	C
Genetta genetta	MNHN-ZM-MO 1994-597		Viverridae	30.96	65.13	20.61	12.26	F	W
Genetta genetta	MNHN-ZM-MO 1997-450	Morphosource Media ID 000166833 (provided by Margot Michaud)	Viverridae	31.09	64.45	20.04	11.93	U	U
Genetta johnstoni	MNHN-ZM-MO 1982-1007		Viverridae	30.30	70.45	23.21	13.83	U	W
Genetta johnstoni	MNHN-ZM-MO 2000-683		Viverridae	30.93	62.29	20.33	11.92	U	W
Genetta johnstoni	MNHN-ZM-MO 2001-523		Viverridae	32.79	70.17	22.61	14.85	M	W
Genetta maculata	MNHN-ZM-MO 1907-586		Viverridae	29.52	66.97	20.31	11.45	U	U
Genetta maculata	MNHN-ZM-MO 1974-203		Viverridae	31.25	68.93	22.03	13.52	U	U
Genetta pardina	MNHN-ZM-MO 1977-704		Viverridae	30.56	65.37	21.47	12.60	F	W
Genetta pardina	MNHN-ZM-MO 1977-711		Viverridae	31.89	83.61	23.59	15.75	M	W
Genetta piscivora	RMCA RG30844		Viverridae	29.83	68.72	20.07	11.54	U	U
Genetta poensis	MNHN-ZM-MO 1894-263		Viverridae	32.93	71.70	23.32	15.52	F	U
Genetta poensis	MNHN-ZM-MO 1897-508		Viverridae	31.98	73.29	21.57	13.86	U	C
Genetta servalina	MNHN-ZM-MO 1885-840		Viverridae	29.36	65.46	20.65	11.50	U	W
Genetta servalina	MNHN-ZM-AC 1983-15		Viverridae	30.97	68.49	19.74	11.88	M	U
Genetta thierryi	MNHN-ZM-MO 1995-420		Viverridae	29.35	60.74	19.80	10.77	F	W
Genetta thierryi	MNHN-ZM-MO 1995-423		Viverridae	28.90	57.06	19.20	10.04	M	W
Gulo gulo	MNHN-ZM-MO 1983-946		Mustelidae	62.92	111.26	39.81	70.43	F	C
Gulo gulo	MNHN-ZM-MO 1995-1208		Mustelidae	70.11	118.84	39.17	80.91	U	U
Helarctos malayanus	MNHN-ZM-AC 1914-360		Ursidae	98.26	169.24	62.50	225.05	U	U
Helarctos malayanus	MNHN-ZM-AC 1971-188		Ursidae	84.31	148.09	56.61	160.70	U	U
Helogale parvula	MNHN-ZM-MO 1987-176		Herpestidae	23.40	42.67	15.82	5.77	F	C
Herpailurus yagouaroundi	MNHN-ZM-MO 1998-240		Felidae	46.99	81.67	33.28	36.88	U	W
Herpailurus yagouaroundi	MNHN-ZM-AC 1920-216		Felidae	52.23	85.16	33.28	42.67	F	C
Herpestes ichneumon	MNHN-ZM-MO 2003-128		Herpestidae	34.20	79.83	25.68	18.59	U	U
Herpestes ichneumon	MNHN-ZM 2005-855		Herpestidae	34.88	81.04	25.48	18.99	U	U
Hyaena hyaena	MNHN-ZM-AC 1910-90		Hyaenidae	67.43	119.31	55.80	112.17	M	C
Hyaena hyaena	MNHN-ZM-AC 1930-220		Hyaenidae	70.94	136.14	54.87	121.95	U	U
Hyaena hyaena	OM 9001	African fossils	Hyaenidae	72.25	138.39	48.50	110.02	U	U
Hyaena hyaena	USNM 182034	Digimorph (provided by Blaire Van Valkenburgh)	Hyaenidae	67.17	149.70	46.82	98.76	M	W
Ichneumia albicauda	MNHN-ZM-MO 1938-848		Herpestidae	37.54	80.04	27.47	22.49	F	U
Ichneumia albicauda	MNHN-ZM-MO 1939-185		Herpestidae	39.77	101.91	28.52	26.95	U	U
Ichneumia albicauda	MNHN-ZM-MO 1981-667		Herpestidae	37.83	83.50	25.77	21.48	F	W
Ichneumia albicauda	MNHN-ZM-MO 2001-2187		Herpestidae	38.55	75.81	28.73	24.03	M	W

Ictonyx libyca	MNHN-ZM-MO 1943-146		Mustelidae	25.62	38.24	15.73	6.24	M	W
Ictonyx libyca	MNHN-ZM-MO 1961-981		Mustelidae	27.16	41.93	17.58	7.75	U	U
Ictonyx striatus	MNHN-ZM-MO 1949-515		Mustelidae	27.43	49.21	20.02	9.42	U	C
Ictonyx striatus	MNHN-ZM-MO 2001-2185		Mustelidae	27.15	50.22	20.55	9.62	M	U
Leopardus colocolo	MNHN-ZM-MO 1897-1261		Felidae	44.77	76.53	24.92	25.06	F	U
Leopardus geoffroyi	MNHN-ZM-MO 1935-356		Felidae	46.88	87.10	30.81	34.49	U	W
Leopardus jacobitus	MNHN-ZM 2006-546		Felidae	39.36	62.49	24.27	19.55	U	W
Leopardus pardalis	MNHN-ZM-AC 1873-258		Felidae	54.51	100.93	36.56	52.19	F	U
Leopardus pardalis	MNHN-ZM-MO 1981-1244		Felidae	55.92	114.98	40.06	61.60	F	C
Leopardus pardalis	MNHN-ZM 2013-33		Felidae	59.14	103.09	39.75	63.63	U	U
Leopardus wiedii	MNHN-ZM-MO 1930-486		Felidae	50.69	84.74	34.14	42.14	U	U
Leopardus wiedii	MNHN-ZM-MO 1962-2954		Felidae	50.43	85.17	31.48	38.47	U	U
Leptailurus serval	MNHN-ZM-AC 1924-397		Felidae	54.41	96.56	37.11	52.25	U	W
Leptailurus serval	MNHN-ZM-AC 1963-75	Morphosource Media ID 000166837 (provided by Margot Michaud)	Felidae	53.64	102.35	33.32	46.53	M	U
Leptailurus serval	MNHN-ZM-MO 1995-452		Felidae	56.34	96.74	35.33	51.87	F	W
Lontra canadensis	MNHN-ZM 2005-604		Mustelidae	52.36	76.33	34.66	43.34	U	U
Lontra canadensis	MNHN-ZM 2005-609		Mustelidae	57.79	70.62	33.69	46.63	U	U
Lontra felina	MNHN-ZM-MO 1932-3019		Mustelidae	49.79	69.06	28.44	66.48	M	U
Lontra felina	MNHN-ZM-AC 1995-185		Mustelidae	57.11	82.09	38.48	55.17	F	W
Lontra longicaudis	MNHN-ZM-MO 2001-1291		Mustelidae	54.15	79.17	35	46.17	F	W
Lontra longicaudis	MNHN-ZM-MO 2001-1970		Mustelidae	65.41	97.90	39.75	71.27	M	W
Lupulella adusta	MNHN-ZM-MO 1934-736		Canidae	50.59	95.29	37.02	47.34	U	U
Lupulella adusta	MNHN-ZM-MO 1972-389		Canidae	48.19	87.84	34.64	40.54	F	W
Lupulella mesomelas	MNHN-ZM-MO 1939-167		Canidae	49.57	85.07	35.31	42.50	M	W
Lupulella mesomelas	MNHN-ZM 2007-433		Canidae	53.22	85.71	40.45	53.83	U	W
Lutra lutra	MNHN-ZM-MO 1996-2466		Mustelidae	55.81	87.40	38.52	54.59	M	U
Lutra lutra	MNHN-ZM 2005-597		Mustelidae	60.10	89.72	39.37	61.84	U	W
Lutra lutra	MNHN-ZM 2005-599		Mustelidae	53.27	84.58	35.77	47.13	U	U
Lutrogale perspicillata	MNHN-ZM-MO 1882-2947		Mustelidae	52.20	80.51	36.67	46.52	U	W
Lutrogale perspicillata	MNHN-ZM-MO 1962-1646		Mustelidae	63.54	75.94	37.41	60.00	F	W
Lycalopex culpaeus	MNHN-ZM-MO 1897-1252		Canidae	52.02	90.75	38.80	50.84	M	W
Lycalopex culpaeus	MNHN-ZM-MO 1962-1522		Canidae	52.36	86.79	42.29	55.47	M	W
Lycalopex griseus	MNHN-ZM-MO 1883-154		Canidae	42.96	71.83	29.09	27.52	U	U
Lycalopex griseus	MNHN-ZM 2007-463		Canidae	50.75	82.64	34.91	42.91	U	W
Lycaon pictus	MNHN-ZM-MO 1962-1528		Canidae	75.80	124.25	58.49	138.37	U	U
Lyncodon patagonicus	MNHN-ZM-AC 1897-422		Mustelidae	21.95	34.69	13.39	4.20	U	U
Lyncodon patagonicus	MNHN-ZM 2005-635		Mustelidae	25.50	40.94	16.87	6.80	U	U
Lynx canadensis	MNHN-ZM-AC 1957-122		Felidae	56.84	95.77	35.75	52.96	F	U
Lynx canadensis	MNHN-ZM-MO 1976-332		Felidae	57.69	101.97	37.97	58.55	M	C
Lynx canadensis	MNHN-ZM 2006-535		Felidae	57.84	92.31	35.87	53.78	M	W
Lynx canadensis	MNHN-ZM 2006-536		Felidae	54.75	98.20	36.59	52.13	U	W
Lynx lynx	MNHN-ZM-MO 1941-79		Felidae	60.76	109.87	39.27	66.18	U	C
Lynx lynx	MNHN-ZM-MO 1997-442		Felidae	66.69	105.82	45.34	85.83	F	W
Lynx rufus	MNHN-ZM-MO 1997-445		Felidae	56.47	97.39	35.14	51.82	M	W
Lynx rufus	MNHN-ZM 2004-303		Felidae	52.58	93.19	32.28	42.72	U	U
Martes americana	MNHN-ZM-MO 1987-422		Mustelidae	31.91	52.96	18.33	10.62	F	W
Martes americana	MNHN-ZM 2005-613		Mustelidae	36.98	61.42	21.14	15.52	F	U
Martes flavigula	MNHN-ZM-MO 1870-47		Mustelidae	44.07	72.76	26.02	25.35	U	W
Martes flavigula	MNHN-ZM-MO 1929-405		Mustelidae	40.86	68.87	23.54	20.39	F	U
Martes foina	MNHN-ZM-MO 1994-808		Mustelidae	38.55	65.75	25.40	20.26	M	W
Martes martes	MNHN-ZM-MO 1994-206		Mustelidae	36.48	65.59	21.83	16.08	M	U
Martes martes	MNHN-ZM 2005-232		Mustelidae	36.73	66.35	23.56	17.64	M	U
Martes zibellina	MNHN-ZM-MO 1872-294		Mustelidae	34.91	57.25	19.38	12.90	U	W
Martes zibellina	MNHN-ZM-MO 1902-1026		Mustelidae	37.03	66.60	21.63	16.30	M	U
Meles meles	MNHN-ZM-AC 1917-131		Mustelidae	47.04	91.70	32.23	36.86	M	U
Meles meles	MNHN-ZM-AC 1987-28		Mustelidae	47.82	89.65	39.34	46.20	F	C
Meles meles	MNHN-ZM 2005-707		Mustelidae	51.96	103.38	41.78	56.95	M	W
Mellivora capensis	MNHN-ZM 2004-453		Mustelidae	62.48	101.20	43.54	74.75	M	W
Melogale moschata	MNHN-ZM-AC 1892-1015		Mustelidae	30.81	51.14	21.09	11.67	F	C
Melogale personata	MNHN-ZM-MO 1923-386		Mustelidae	24.70	52.94	18.50	7.74	U	U
Melogale personata	MNHN-ZM-MO 1929-374		Mustelidae	31.48	49.76	20.75	11.70	F	W
Melursus ursinus	MNHN-ZM-AC 1883-59		Ursidae	96.58	209.56	70.16	264.22	U	U
Mephitis mephitis	USNM 260921		Mephitidae	28.99	46.79	20.15	10.04	F	U
Mephitis mephitis	USNM 564277		Mephitidae	28.08	50.93	21.48	10.56	F	U
Mungos mungo	MNHN-ZM_AC 1894-414		Herpestidae	28.78	53.14	19	9.68	U	C
Mungos mungo	MNHN-ZM-MO 1897-636		Herpestidae	27.46	52	18.46	8.79	M	U
Mungos mungo	MNHN-ZM-MO 1904-2017		Herpestidae	28.32	53.98	18.84	9.44	U	W
Mungos mungo	MNHN-ZM-MO 1962-2075		Herpestidae	29.35	55.38	19.32	10.22	U	U
Mungotictis decemlineata	MNHN-ZM-MO 1992-1670		Eupleridae	27.62	50.02	17.05	8.06	M	C
Mungotictis decemlineata	NHMUK 48.212		Eupleridae	25.57	45.63	16.79	7.00	F	U
Mungotictis decemlineata	NHMUK 48.2121		Eupleridae	25.74	43.13	17.49	7.26	F	NA
Mustela erminea	MNHN-ZM-MO 1944-444		Mustelidae	20.35	36.47	12.65	3.65	U	U
Mustela erminea	MNHN-ZM-MO 1995-1697		Mustelidae	20.48	35.13	12.49	3.59	M	W
Mustela eversmanni	MNHN-ZM 2005-668		Mustelidae	29.26	50.10	18.93	9.69	M	W
Mustela frenata	MNHN-ZM-MO 1932-2857		Mustelidae	21.98	41.25	14.79	4.91	U	U
Mustela frenata	MNHN-ZM 2005-625		Mustelidae	18.98	35.18	12.92	3.38	M	W
Mustela lutreola	MNHN-ZM-AC 1962-305		Mustelidae	28.41	50.10	18.79	9.26	M	U
Mustela lutreola	MNHN-ZM-AC 1962-332		Mustelidae	28.88	49.90	19.58	9.86	F	U
Mustela nivalis	MNHN-ZM-AC 1927-89		Mustelidae	14.54	29.65	10.09	1.77	F	W

Mustela nivalis	MNHN-ZM-MO 2003-483		Mustelidae	20.16	35.03	12.66	3.57	U	U
Mustela putorius	MNHN-ZM-MO 1991-605		Mustelidae	29.28	50.26	18.50	9.47	F	W
Mustela putorius	MNHN-ZM-MO 1992-2003		Mustelidae	32.73	52.99	19.14	11.48	F	U
Mustela putorius	MNHN-ZM 2004-639		Mustelidae	30.34	52.06	19.08	10.34	U	W
Mustela sibirica	MNHN-ZM-MO 1886-983		Mustelidae	24.79	52.07	16.34	6.79	U	W
Mustela sibirica	MNHN-ZM-MO 1962-1628		Mustelidae	27.71	50.32	18.31	8.74	M	W
Nandinia binotata	MNHN-ZO-MO_1958-722		Nandiniidae	33.18	67.33	22.26	14.66	U	C
Nandinia binotata	MNHN-ZO-MO_1966-213		Nandiniidae	32.17	63.39	22.19	13.81	U	W
Nandinia binotata	MNHN-ZM 2005-806		Nandiniidae	32.54	65.67	22.04	14.05	U	U
Nasua narica	MNHN-ZM-AC 1918-10		Procyonidae	43.12	74.72	33.71	32.69	F	C
Nasua narica	MNHN-ZM-AC 1934-554		Procyonidae	42.41	68.02	28.49	26.08	U	C
Nasua narica	MNHN-ZM 2005-869		Procyonidae	44.76	79.41	30.53	31.39	U	U
Nasua nasua	MNHN-ZM-MO 1956-706		Procyonidae	47.72	82.39	33.52	37.98	F	W
Nasua nasua	MNHN-ZM-MO 1977-48		Procyonidae	42.52	76.32	28.78	27.32	M	C
Nasua nasua	MNHN-ZM 2010-640		Procyonidae	44.20	80.79	34.87	35.73	F	C
Nasuella olivacea	MNHN-ZM-MO 1929-598		Procyonidae	39.95	70.53	30.09	25.88	F	W
Neofelis nebulosa	MNHN-ZM-AC 1940-305		Felidae	50.14	113.49	38.92	51.83	U	W
Neofelis nebulosa	MNHN-ZO-MO 1960-3663		Felidae	58.84	126.13	126.13	68.00	M	C
Neofelis nebulosa	MNHN-ZM 2006-430		Felidae	63.35	132.52	40.60	76.20	M	W
Neovison vison	MNHN-ZM-MO 1958-165		Mustelidae	27.52	47.79	19.77	9.26	M	W
Neovison vison	MNHN-ZM 2005-646		Mustelidae	27.09	42.03	16.97	7.45	F	W
Nyctereutes procyonoides	MNHN-ZM-MO 1874-485		Canidae	41.94	65.60	27.65	24.66	U	W
Nyctereutes procyonoides	MNHN-ZM-MO 1977-44		Canidae	40.24	65.85	27.24	23.06	M	W
Otocolobus manul	MNHN-ZM 2009-251		Felidae	46.95	67.79	27.21	28.24	F	C
Otocolobus manul	MNHN-ZM 2010-646		Felidae	47.35	68.45	25.51	26.73	M	C
Otocyon megalotis	MNHN-ZM-MO 1972-392		Canidae	43.44	59.84	26.45	23.98	M	W
Otocyon megalotis	MNHN-ZM-MO 1973-136		Canidae	42.73	59.55	28.08	24.98	M	U
Otocyon megalotis	USNM 429129	Morphosource Media ID 000100989 (provided by Blaire Van Valkenburgh & Tim Rowe)	Canidae	44.45	63.50	28.66	27.33	M	W
Otocyon megalotis	USNM 429132	Digimorph (provided by Blaire Van Valkenburgh)	Canidae	45.16	75.07	28.13	28.65	F	W
Paguma larvata	MNHN-ZM-MO 1962-1588		Viverridae	38.30	72.62	26.32	21.46	M	U
Paguma larvata	MNHN-ZM-MO 1962-2062		Viverridae	40.02	77.93	29.10	25.74	U	C
Panthera leo	MNHN-ZM-AC 1938-277		Felidae	84.16	176.76	84.70	258.26	M	C
Panthera leo	MNHN-ZM-AC 1938-632		Felidae	94.65	191.33	66.49	237.16	M	C
Panthera leo	MNHN-ZM-MO 1974-312		Felidae	88.41	183.37	64.86	209.33	M	C
Panthera leo	MNHN-ZM-AC 1995-164		Felidae	92.84	167.41	67.69	227.19	U	U
Panthera leo	MVZ 117849	Digimorph (provided by Blaire Van Valkenburgh & Jessica Theodor)	Felidae	103.82	227.98	73.55	311.75	U	W
Panthera leo	NA	African fossils	Felidae	83.20	170.33	81.23	240.97	U	U
Panthera onca	MNHN-ZM-AC 1914-259		Felidae	76.14	128.16	50.31	119.65	F	C
Panthera onca	MNHN-ZM 2006-438		Felidae	82.28	154.91	69.25	195.36	U	U
Panthera pardus	AMNH M-113745	Morphosource Media ID 000009401 (provided by Jack Tseng)	Felidae	80.98	163.55	47.99	131.77	U	U
Panthera pardus	MNHN-ZM-MO 1962-988		Felidae	82.78	154.70	50.84	141.83	U	W
Panthera tigris	MNHN-ZM-AC 1931-60		Felidae	89.28	179.56	73.37	240.12	U	U
Panthera tigris	MNHN-ZM-AC 1983-119		Felidae	95.32	184.85	71.14	254.60	F	C
Panthera uncia	MNHN-ZM-AC 1970-102		Felidae	76.62	119.85	44.86	104.82	M	C
Panthera uncia	MNHN-ZM-AC 1979-52		Felidae	92.05	146.21	58.14	184.16	U	U
Panthera uncia	MNHN-ZM 2006-429		Felidae	75.10	122.53	39.43	89.68	U	W
Paradoxurus hermaphroditus	MNHN-ZM-MO 1982-164		Viverridae	37.86	78.97	26.60	21.89	F	W
Paradoxurus hermaphroditus	MNHN-ZM-MO 1990-581		Viverridae	33.50	63.15	21.83	14.28	F	W
Paradoxurus hermaphroditus	MNHN-ZM-MO A3448	Morphosource Media ID 000166841 (provided by Margot Michaud)	Viverridae	35.72	72.66	24.47	18.18	U	U
Paradoxurus zeylonensis	MNHN-ZM-MO 1876-2018		Viverridae	34.14	70.27	21.79	15.04	U	W
Parahyaena brunnea	FMNH 38584	Digimorph (provided by Blaire Van Valkenburgh)	Hyaenidae	79.83	156.86	50.30	134.43	M	W
Pardofelis marmorata	MNHN-ZM-MO 1886-25		Felidae	44.90	79.63	28.44	29.25	U	W
Poecilogale albinucha	MNHN-ZM-AC 1934-107		Mustelidae	30.84	48.47	20.77	11.32	U	U
Poecilogale albinucha	MNHN-ZM-MO 1981-1371		Mustelidae	29.88	51.45	22.02	11.77	U	W
Poiana richardsonii	MNHN-ZM-MO-1976-389		Viverridae	25.60	50.28	14.09	5.98	U	W
Poiana richardsonii	RMCA 75-30-M-25		Viverridae	25.58	48.75	15.03	6.35	U	W
Potos flavus	MNHN-ZM-MO 1995-956		Procyonidae	36.85	63.27	24.28	18.05	M	W
Potos flavus	MNHN-ZM-MO 1995-957		Procyonidae	38.63	65.24	25.54	20.39	M	W
Potos flavus	MNHN-ZM-MO 2000-689		Procyonidae	38.58	68.71	24.33	19.62	F	W
Prionailurus bengalensis	MNHN-ZM-MO 1874-494		Felidae	35.63	61.49	24.28	17.16	U	U
Prionailurus planiceps	MNHN-ZM-MO 1873-228		Felidae	38.66	75.32	28.25	23.65	M	W
Prionailurus viverrinus	MNHN-ZM-MO 1953-866		Felidae	50.19	102.72	42.26	55.07	U	W
Prionailurus viverrinus	MNHN-ZM 2015-1300		Felidae	45.67	86.49	29.99	32.36	F	C
Prionailurus viverrinus	MNHN-ZM-AC A12519		Felidae	49.97	104.76	42.09	54.83	U	U
Prionodon linsang	USNM 303036	Digimorph (provided by Blaire Van Valkenburgh & Tim Rowe)	Prionodontidae	23.87	53.59	15.59	6.21	M	W
Prionodon pardicolor	MNHN-ZM-MO 1929-424		Prionodontidae	22.98	49.06	14.38	5.30	U	U
Procyon cancrivorus	MNHN-ZM-MO 1955-596		Procyonidae	58.53	79.69	36.11	52.77	U	W
Procyon cancrivorus	MNHN-ZM-MO 2000-364		Procyonidae	54.81	90.06	36.56	50.90	M	W

Procyon cancrivorus	MNHN-ZM-MO 2000-828		Procyonidae	56.18	96.36	35.44	51.79	M	W
Procyon lotor	MNHN-ZM-AC 1871-287		Procyonidae	44.60	77.26	27.10	27.33	U	U
Procyon lotor	MNHN-ZM-AC 1934-556		Procyonidae	50.82	85.04	27.97	34.26	U	C
Procyon lotor	MNHN-ZM-MO 1948-512		Procyonidae	49.49	94.05	30.04	36.75	U	C
Profelis aurata	MNHN-ZM-MO 1917-18		Felidae	52.09	102.68	35	47.27	U	U
Profelis aurata	RMCA 18469		Felidae	55.92	107.55	38.39	57.79	U	W
Proteles cristata	LACM 60619	Phenome10k<br>(provided by Jack Tseng)	Hyaenidae	55.93	94.03	31.05	44.46	U	U
Pteronura brasiliensis	MNHN-ZM-AC A1918		Mustelidae	69.85	123.30	52.23	110.38	M	W
Puma concolor	MNHN-ZM-AC 1893-241		Felidae	76.30	135.35	54.34	132.19	M	C
Puma concolor	MNHN-ZM-MO 1973-2		Felidae	70.96	130.15	55.15	121.12	U	U
Puma concolor	MNHN-ZM-MO 1993-4625		Felidae	71.54	125.77	55.33	121.63	F	C
Rhynchogale melleri	MNHN-ZM-MO 1962-992		Herpestidae	34.84	72.80	25.96	18.76	M	U
Rhynchogale melleri	RMCA 21-881		Herpestidae	33	66.89	25.45	16.75	M	W
Salanoia concolor	MNHN-ZM-MO 1866-233		Eupleridae	26.58	48.15	18.03	8.05	F	U
Salanoia concolor	MNHN-ZM-MO 1962-2107		Eupleridae	27.27	49.10	19.16	8.92	U	W
Salanoia concolor	MNHN-ZM-MO 1962-2108		Eupleridae	28.13	52.15	19.40	9.57	U	W
Salanoia concolor	MNHN-ZM-MO 1962-2110		Eupleridae	27.81	48.96	18.84	8.98	U	W
Salanoia concolor	MNHN-ZM-MO 1962-2111		Eupleridae	28.60	50.57	18.24	9.07	U	W
Speothos venaticus	MNHN-ZM-MO 1998-588		Canidae	47.19	91.87	38.35	44.53	M	C
Speothos venaticus	MNHN-ZM-AC 2000-369		Canidae	46.40	92.46	40.14	45.82	M	C
Spilogale gracilis	MNHN-ZM 2005-656		Mephitidae	23.29	37.36	15.70	5.48	U	W
Spilogale putorius	MNHN-ZM 2005-657		Mephitidae	24.22	37.93	17.33	6.42	U	W
Spilogale putorius	USNM 564280		Mephitidae	23.41	35.91	15.04	5.21	F	U
Suricata suricatta	MNHN-ZM-MO 1936-1534		Herpestidae	29.94	43.11	19.48	9.86	F	U
Suricata suricatta	MNHN-ZM-MO 1998-112		Herpestidae	31.99	50.66	21.44	12.42	F	C
Taxidea taxus	LACM 45012	Morphosource Media ID<br>000100743<br>(provided by Blaire Van<br>Valkenburgh & Tim Rowe)	Mustelidae	56.87	74.74	36.51	50.56	F	W
Taxidea taxus	NMB 9186		Mustelidae	55.52	74.61	36.88	49.54	F	U
Taxidea taxus	NMB 9783		Mustelidae	56.78	74.85	40.58	56.46	M	U
Tremarctos ornatus	MNHN-ZM-AC 1990-696		Ursidae	87.14	156.08	50.68	151.25	F	C
Tremarctos ornatus	MNHN-ZM-AC 1992-1469		Ursidae	91.50	161.73	56.11	181.05	F	C
Urocyon cinereoargenteus	MNHN-ZM-MO 1898-1484		Canidae	42.05	73.14	26.05	23.95	U	U
Ursus americanus	MNHN-ZM-MO 1938-125		Ursidae	89.92	162.77	63.34	201.73	U	W
Ursus americanus	USNM 227070	Digimorph<br>(provided by Blaire Van<br>Valkenburgh)	Ursidae	95.20	194.22	73.94	268.50	U	W
Ursus arctos	MNHN-ZM-AC 1963-400		Ursidae	110.22	212.22	82.16	370.73	F	C
Ursus arctos	MNHN-ZM-AC 2000-482		Ursidae	116.11	238.44	70.19	346.30	F	C
Ursus arctos	USNM 98062	Digimorph<br>(provided by Tim Rowe)	Ursidae	103.87	220.96	65.85	275.02	F	W
Ursus maritimus	MNHN-ZM-MO 1996-2172		Ursidae	99.90	217.44	96.95	392.64	F	U
Ursus maritimus	USNM 275072	Digimorph<br>(provided by Blaire Van<br>Valkenburgh)	Ursidae	111.46	265.67	105.66	522.81	U	W
Ursus thibetanus	MNHN-ZM-MO 1964-244		Ursidae	94.63	160.55	66.28	224.98	F	C
Ursus thibetanus	MNHN-ZM-MO 1986-345		Ursidae	108.20	197.20	75.13	322.66	U	W
Urva brachyura	MNHN-ZM-MO 1872-278		Herpestidae	35.75	72.97	24.72	18.42	U	W
Urva brachyura	MNHN-ZM-MO 1965-406		Herpestidae	37.87	82.26	26.45	22.02	F	C
Urva brachyura	MNHN-ZM-MO 1974-309		Herpestidae	37.08	72.89	25.18	19.67	U	U
Urva edwardsii	MNHN-ZM 2005-854		Herpestidae	26.52	57.77	18.81	8.84	U	U
Urva edwardsii	MNHN-ZM-AC A3444		Herpestidae	27.11	59.09	20.46	10.00	F	C
Urva javanica	MNHN-ZM 2004-621		Herpestidae	24.33	49.65	19.18	7.75	U	U
Urva javanica	MNHN-ZM 2004-622		Herpestidae	24.89	52.38	17.99	7.57	U	U
Urva urva	MNHN-ZM-MO 1929-380		Herpestidae	39.65	74.09	26.35	22.58	M	W
Viverra tangalunga	MNHN-ZM-MO 1884-11		Viverridae	35.42	70.58	26.23	19.21	M	W
Viverra tangalunga	MNHN-ZM-AC 1884-2464		Viverridae	34.99	70.48	25.65	18.46	F	W
Viverra tangalunga	MNHN-ZM-AC 1884-2478		Viverridae	35.22	66.35	25.70	18.34	M	W
Viverra tangalunga	MNHN-ZM 2005-880		Viverridae	34.76	66.51	26.36	18.54	U	W
Viverra zibetha	MNHN-ZM-AC 1877-703		Viverridae	44.63	93.66	38.67	42.07	U	W
Viverra zibetha	MNHN-ZM-AC A3493		Viverridae	38.25	77.32	27.98	23.26	U	U
Viverricula indica	MNHN-ZM-AC 1951-4		Viverridae	31.10	73.56	22.46	13.97	U	U
Viverricula indica	MNHN-ZM-MO 1962-2113		Viverridae	31.97	65.22	20.55	12.73	U	W
Viverricula indica	MNHN-ZM-MO 1962-2115		Viverridae	31.17	67.41	22.58	13.75	U	W
Viverricula indica	MNHN-ZM 2005-884		Viverridae	31.78	69.02	22.80	14.34	U	W
Vormela peregusna	NMB 8548		Mustelidae	24.38	37.96	17.54	6.56	M	U
Vormela peregusna	NMB 8554		Mustelidae	25.4	34.56	16.14	6.16	F	U
Vulpes lagopus	MNHN-ZM-MO 1962-1549		Canidae	46.41	76.70	32.71	35.01	U	U
Vulpes lagopus	MNHN-ZM 2007-443		Canidae	46.26	68.98	28.93	29.72	U	W
Vulpes rueppellii	MNHN-ZM-MO 1962-143		Canidae	41.34	60.57	23.87	20.26	U	W
Vulpes rueppellii	MNHN-ZM-MO 1994-619		Canidae	40.17	60.18	23.35	19.05	U	W
Vulpes velox	MNHN-ZM-AC 1868-217		Canidae	48.08	83.32	29.47	33.56	U	U
Vulpes vulpes	MNHN-ZM-MO 1962-1544		Canidae	43.34	70.07	28.81	27.36	F	W
Vulpes vulpes	MNHN-ZM-MO 1996-519		Canidae	48.50	82.64	34.83	40.41	U	W
Vulpes zerda	MNHN-ZM-MO 1981-126		Canidae	37.62	53.63	20.02	14.42	U	U
Vulpes zerda	MNHN-ZM-MO 1995-460		Canidae	34.91	51.26	19.35	12.49	U	W
Xenogale naso	MNHN-ZM-MO 1970-20		Herpestidae	37.34	75.30	29.84	23.98	U	U
Xenogale naso	MNHN-ZM-MO 1988-278		Herpestidae	39.04	81.39	28.05	24.28	U	U

Supplementary Data 2. **List of species, number of specimens (N) and associated estimated brain volume, average body mass and phylogenetic encephalization quotient used in this study.** Brain volumes extracted from Finarelli (2006) are indicated with an asterisk.

Species	Family	N	Measured brain volume (ml)	Average body mass (g)	Phylogenetic Encephalization Quotient (EQ)
Acinonyx jubatus	Felidae	2	116.89	50577.92	-0.087606987
Ailuropoda melanoleuca	Ursidae	1	292.98	117999.99	0.310128075
Ailurus fulgens	Ailuridae	3	44.31	5170.08	0.345042830
Amblonyx cinereus	Mustelidae	2	30.01	3527.59	0.190584825
Aonyx capensis	Mustelidae	1	76.63*	806.21	0.081988383
Arctictis binturong	Viverridae	2	39.92	12999.99	-0.326398566
Arctogalidia trivirgata	Viverridae	2	17.39	2323.79	-0.098183063
Arctonyx collaris	Mustelidae	2	59.00	8166.52	0.350338862
Atelocynus microtis	Canidae	9	57.43*	8363.22	0.308652532
Atilax paludinosus	Herpestidae	3	23.04	3600.16	-0.086175173
Bassaricyon alleni	Procyonidae	3	12.21	1235.01	-0.063003327
Bassaricyon gabbii	Procyonidae	1	12.58	1250	-0.040768872
Bassariscus astutus	Procyonidae	3	12.06	1010.37	0.048080723
Bassariscus sumichrasti	Procyonidae	2	18.72*	906	0.554757891
Bdeogale crassicauda	Herpestidae	2	14.85	1722.13	-0.071611562
Bdeogale nigripes	Herpestidae	1	26.28	2623.01	0.240044954
Canis aureus	Canidae	2	50.05	9658.7	0.082498368
Canis latrans	Canidae	2	73.05	11989.1	0.327646011
Canis lupus	Canidae	4	123.20	31756.51	0.251171707
Canis simensis	Canidae	2	87.20	14361.86	0.393717824
Caracal caracal	Felidae	3	49.46	11964.38	-0.060910679
Catopuma temminckii	Felidae	2	49.46	7726.46	0.558383595
Cerdocyon thous	Canidae	1	38.60	5741.66	0.142554644
Chrotogale owstoni	Viverridae	1	20.56	3267.71	-0.140684362
Chrysocyon brachyurus	Canidae	6	97.76*	23325	0.209711756
Civettictis civetta	Viverridae	3	34.91	12075.58	-0.415218564
Conepatus chinga	Mephitidae	2	11.30	1918	-0.411705523
Conepatus leuconotus	Mephitidae	1	18.53	3293.91	-0.249653267
Crocuta crocuta	Hyaenidae	4	185.43	63369.98	0.235131075
Crossarchus obscurus	Herpestidae	4	9.12	1395.09	-0.430418362
Cryptoprocta ferox	Eupleridae	6	35.75	9500	-0.243792020
Cuon alpinus	Canidae	2	91.42	15800	0.382228115
Cynictis penicillata	Herpestidae	6	9.54*	694.41	0.044259207
Cynogale bennettii	Viverridae	1	25.34*	4246.57	-0.092662277
Eira barbara	Mustelidae	1	37.43	4134.99	0.313867802
Enhydra lutris	Mustelidae	2	117.92	27410.93	0.297878643
Eupleres goudotii	Eupleridae	4	14.37	2763.34	-0.395764959
Felis chaus	Felidae	1	33.71	7157.99	-0.128273823
Felis margarita	Felidae	2	21.71	2823.36	0.003966906
Felis silvestris	Felidae	3	25.44	4573.08	-0.134110422
Fossa fossana	Eupleridae	6	15.36	1853.98	-0.083533894
Galerella sanguinea	Herpestidae	3	7.27	543.83	-0.076468488

Galictis cuja	Mustelidae	2	17.06	1000	0.400952344
Galictis vittata	Mustelidae	1	26.72	3200	0.134251146
Galidia elegans	Eupleridae	11	9.50	810	-0.054939878
Galidictis fasciata	Eupleridae	4	9.72	974.995	-0.145933117
Genetta bourloni	Viverridae	2	13.78	1700	-0.138896867
Genetta genetta	Viverridae	4	11.44	1756.17	-0.344433816
Genetta johnstoni	Viverridae	3	13.53	2225	-0.322320778
Genetta maculata	Viverridae	2	12.49	1950	-0.321573910
Genetta pardina	Viverridae	2	14.18	3100	-0.479781652
Genetta piscivora	Viverridae	1	11.54	1648.05	-0.091160457
Genetta poensis	Viverridae	2	14.69	2200	-0.233339481
Genetta servalina	Viverridae	2	11.69	1175.84	-0.076390504
Genetta thierryi	Viverridae	2	10.40	1400	-0.300404061
Gulo gulo	Mustelidae	2	75.67	12792.49	0.323066171
Helarctos malayanus	Ursidae	2	192.87	57075.78	0.338828284
Helogale parvula	Herpestidae	1	5.77	281.83	0.095335744
Hemigalus derbyanus	Viverridae	10	17.74*	4246.57	-0.449224475
Herpailurus yagouaroundi	Felidae	2	39.77	6875	0.061878719
Herpestes brachyurus	Herpestidae	3	20.04	1396.16	0.356757897
Herpestes ichneumon	Herpestidae	2	18.79	2980.02	-0.173919886
Hyaena hyaena	Hyaenidae	4	110.72	35070.51	0.083396064
Hydrictis maculicollis	Mustelidae	6	39.54*	4180.53	0.361907059
Ichneumia albicauda	Herpestidae	4	23.74	3628.4	-0.061183010
Ictonyx libyca	Mustelidae	2	6.99	225.18	0.426416097
Ictonyx striatus	Mustelidae	2	9.52	811.02	-0.053263706
Leopardus colocolo	Felidae	1	25.06	5000	-0.204391530
Leopardus geoffroyi	Felidae	1	34.49	3590	0.318990368
Leopardus jacobitus	Felidae	1	19.55	8133.78	-0.752013683
Leopardus pardalis	Felidae	3	59.14	11880	0.122115283
Leopardus wiedii	Felidae	2	40.30	3270.81	0.532083915
Leptailurus serval	Felidae	3	50.22	11999.96	-0.047724706
Lontra canadensis	Mustelidae	2	44.98	8087.42	0.085047403
Lontra felina	Mustelidae	2	60.82	11195.36	-0.145964911
Lontra longicaudis	Mustelidae	2	58.72	6554.97	0.480715268
Lupulella adusta	Canidae	2	43.94	10392.49	-0.092763975
Lupulella mesomelas	Canidae	2	48.17	8247.3	0.141320810
Lutra lutra	Mustelidae	3	54.52	8868.69	0.220571986
Lutrogale perspicillata	Mustelidae	2	53.26	8912.49	0.194088470
Lycalopex culpaeus	Canidae	2	53.15	8616.24	0.213003535
Lycalopex griseus	Canidae	2	35.21	3000	0.450233507
Lycaon pictus	Canidae	1	138.37	21999.99	0.593083810
Lyncodon patagonicus	Mustelidae	2	5.5	225	0.187425538
Lynx canadensis	Felidae	4	54.35	9682.82	0.163517286
Lynx lynx	Felidae	2	76.00	19300	0.074538715
Lynx rufus	Felidae	2	47.27	6374.47	0.281024500
Martes americana	Mustelidae	2	13.07	873.69	0.217845706
Martes flavigula	Mustelidae	2	22.87	2504.64	0.129549713
Martes foina	Mustelidae	1	20.26	1675	0.256032227
Martes martes	Mustelidae	2	16.86	1299.99	0.228048974
Martes pennanti	Mustelidae	10	30.25*	3750	0.160940028

Martes zibellina	Mustelidae	2	14.6	1173.91	0.146859277
Meles meles	Mustelidae	3	46.67	11884.03	-0.114846753
Mellivora capensis	Mustelidae	1	74.75	8999.99	0.527093856
Melogale moschata	Mustelidae	1	11.67	938.5	0.060249148
Melogale personata	Mustelidae	2	9.72	1845.26	-0.538247930
Melursus ursinus	Ursidae	1	264.22	99999.99	0.308640205
Mephitis mephitis	Mephitidae	2	10.3	2399.99	-0.642015442
Mungos mungo	Herpestidae	4	9.53	1260	-0.322713772
Mungotictis decemlineata	Eupleridae	3	7.44	657.03	-0.170400363
Mustela altaica	Mustelidae	7	3.9*	180.24	-0.020637387
Mustela erminea	Mustelidae	2	3.62	284.5	-0.376734588
Mustela eversmanni	Mustelidae	1	9.69	1684.21	-0.485279777
Mustela frenata	Mustelidae	2	4.14	190.03	0.007704474
Mustela lutreola	Mustelidae	2	9.56	566.44	0.171854795
Mustela nigripes	Mustelidae	4	9.32*	907.1	-0.143418300
Mustela nivalis	Mustelidae	2	2.67	78.45	0.110525491
Mustela putorius	Mustelidae	3	10.43	975.55	-0.075553350
Mustela sibirica	Mustelidae	2	7.76	530.85	0.003388921
Mydaus javanensis	Mephitidae	2	16.1*	2500	-0.220338332
Nandinia binotata	Nandiniidae	3	14.17	2167.2	-0.259765853
Nasua narica	Procyonidae	3	30.05	4578.43	0.031601627
Nasua nasua	Procyonidae	3	33.68	3775.5	0.264104614
Nasuella olivacea	Procyonidae	1	25.88	1339.99	0.637855380
Neofelis nebulosa	Felidae	3	65.34	14945.05	0.080602569
Neovison vison	Mustelidae	2	8.35	945	-0.277895696
Nyctereutes procyonoides	Canidae	2	23.86	4214.99	-0.148320069
Otocolobus manul	Felidae	2	27.48	3050	0.192189678
Otocyon megalotis	Canidae	4	26.23	4098.12	-0.036064791
Paguma larvata	Viverridae	2	23.60	4300	-0.171473554
Panthera leo	Felidae	6	247.44	158623.93	-0.040763059
Panthera onca	Felidae	2	157.50	83943.09	-0.101036593
Panthera pardus	Felidae	2	136.80	52399.99	0.047875941
Panthera tigris	Felidae	2	247.36	161914.66	-0.053722720
Panthera uncia	Felidae	3	126.22	32500	0.261186084
Paradoxurus hermaphroditus	Viverridae	3	18.12	3200	-0.254113892
Paradoxurus zeylonensis	Viverridae	1	15.04	2821.3	-0.362835242
Parahyaena brunnea	Hyaenidae	1	134.43	42977.93	0.152331980
Pardofelis marmorata	Felidae	2	30.16	2826.68	0.332101887
Poecilogale albinucha	Mustelidae	2	11.54	308.16	0.734776354
Poiana richardsonii	Viverridae	2	6.16	570.08	-0.270831000
Potos flavus	Procyonidae	3	19.35	2441.81	-0.021812646
Prionailurus bengalensis	Felidae	1	17.16	2780.97	-0.222136637
Prionailurus planiceps	Felidae	1	23.65	3533.76	-0.048773098
Prionailurus viverrinus	Felidae	3	47.42	8826.46	0.083919447
Prionodon lisang	Prionodontidae	1	6.21	684.83	-0.377250075
Prionodon pardicolor	Prionodontidae	1	5.30	1142.68	-0.850779069
Procyon cancrivorus	Procyonidae	3	51.82	6931.72	0.321386212
Procyon lotor	Procyonidae	3	32.78	6373.72	-0.085008380
Profelis aurata	Felidae	2	52.53	11277.17	0.035592934
Proteles cristata	Hyaenidae	1	44.46	8139.39	0.069389919

Pseudalopex gymnocercus	Canidae	3	37.7*	4542.67	0.263154349
Pteronura brasiliensis	Mustelidae	1	110.38	26000	0.264369968
Puma concolor	Felidae	3	124.98	53954.05	-0.060479761
Rhynchogale melleri	Herpestidae	2	17.75	2240.33	-0.054927739
Salanoia concolor	Eupleridae	5	8.92	711.49	-0.038040081
Speothos venaticus	Canidae	2	45.17	6324.54	0.240478936
Spilogale gracilis	Mephitidae	1	5.48	466.5	-0.265908565
Spilogale putorius	Mephitidae	2	5.815	566.49	-0.325628068
Suricata suricatta	Herpestidae	2	11.14	729.99	0.168521723
Taxidea taxus	Mustelidae	3	52.19	7842.15	0.252505587
Tremarctos ornatus	Ursidae	2	166.15	123176.97	-0.283501601
Urocyon littoralis	Canidae	1	27.66*	1923	0.482232621
Urocyon cinereoargenteus	Canidae	1	23.95	3833.71	-0.086300115
Ursus americanus	Ursidae	2	235.11	110500	0.130499202
Ursus arctos	Ursidae	3	330.68	196287.5	0.118172163
Ursus maritimus	Ursidae	2	457.72	371703.81	0.050536997
Ursus thibetanus	Ursidae	2	273.82	99714.19	0.346073195
Urva edwardsii	Herpestidae	2	9.42	1304.67	-0.356341102
Urva javanica	Herpestidae	2	7.66	750	-0.222563027
Urva smithii	Herpestidae	1	12.99*	1702.5	-0.198663553
Urva urva	Herpestidae	1	22.58	2384.19	0.146967317
Viverra tangalunga	Viverridae	4	18.64	7349.99	-0.737324745
Viverra zibetha	Viverridae	2	32.66	9148.77	-0.310707837
Viverricula indica	Viverridae	4	13.70	2918.88	-0.477126316
Vormela peregusna	Mustelidae	2	6.36	594.13	-0.265351820
Vulpes chama	Canidae	3	33.9*	2919.80	0.428780051
Vulpes lagopus	Canidae	2	32.36	3584.37	0.256352807
Vulpes rueppellii	Canidae	2	19.65	3249.97	-0.182095695
Vulpes velox	Canidae	1	33.56	2088	0.624878887
Vulpes vulpes	Canidae	2	33.88	4820.36	0.119962439
Vulpes zerda	Canidae	2	13.45	1317.13	-0.005751817
Xenogale naso	Herpestidae	2	24.13	3002.08	0.071762630
