# Supplementary material for: The impact of environmental factors on the evolution of brain size in carnivorans
Source: Commun Biol. 2022 Sep 21;5:998. doi: 10.1038/s42003-022-03748-4 (PMC9492690; doi:10.1038/s42003-022-03748-4)
Supplement: Supplementary file 5 — Supplementary Code 1 [file 42003_2022_3748_MOESM5_ESM.pdf]

Supplementary Code 1. The RCode used in this study.

```
## Libraries needed
library(readxl) # import de l'excel
library(dplyr) # manipulation avancée de data frame
library(tidyverse)
library(rcompanion)
library(corrplot)
library(nlme)
library(phytools)
library(caper)
library(ape)
library(fastDummies)
library(ggplot2)
library(gridExtra)
library(rsample)
library(rpart)
library(rpart.plot)
library(treeClust)
library(RRphylo)
library(plotrix)
library(RColorBrewer)
library(ape)
library(phytools)
library(smatr)

## PART 1 ##
## Importe et affiche l'excel du dataset1 (que BM et Body mass sur 174 species)
# ATTENTION: bien changer dans l'excel les points en virgules, car étant un excel français, il prenait les chiffres pour du texte
Ecology_complete_S_all <- read_excel("C:/Users/mmar3/Dropbox/Dossier pro/Projet FYSSEN/Article - Brain mass/Manuscript/V2/Script/brain/Ecology_complete_S_all.xlsx")
Ecology_complete_S_all=data.frame(Ecology_complete_S_all)

## Calcul du EQ et creation d'une colonne dans tableau de data1
# On fait une pgls du log(brainmass)~log(bodymass) et on prend le résidu qui est le EQ. Comme ça on contrôle pour la phylogénie, mais on a une valeur fixe.
#importer la phylogénie
PhyloTree=read.nexus(file="C:/Users/mmar3/Dropbox/Dossier pro/Projet FYSSEN/Article - Brain mass/Manuscript/V2/Script/brain/Phy_Slater.nex")
#plot(PhyloTree,cex=.1)

#creer le comparative dataset pour faire la pgls et calculer EQ
CompData=comparative.data(PhyloTree,data=Ecology_complete_S_all[,c("BM","Mass_Pantheria","Species")],names.col="Species")

#faire la pgls pour calculer le EQ
modelEQ=pgls(log(BM)~log(Mass_Pantheria),data=CompData,lambda="ML")
Ecology_complete_S_all$EQsev<-modelEQ$residuals
summary(modelEQ)
```

```
#On le fait aussi en version non phylogénétique pour voir les différences potentielles dans la suite des analyses
modelEQ2=gls(log(BM)~log(Mass_Pantheria),data=Ecology_complete_S_all)
Ecology_complete_S_all$EQsev2<-modelEQ2$residuals
summary(modelEQ2)

### Plot avec la couleur
theme<-theme(panel.background = element_blank(),panel.border=element_rect(fill=NA),panel.grid.major = element_blank(),panel.grid.minor = element_blank(),strip.background=element_blank(),axis.text.x=element_text(colour="black"),axis.text.y=element_text(colour="black"),axis.ticks=element_line(colour="black"),plot.margin=unit(c(1,1,1,1),"line"))
regplot <- ggplot(Ecology_complete_S_all, aes(x= log(Mass_Pantheria),y=log(BM), color=modelEQ$residuals)) + geom_point(size = 4, shape = 19) + scale_color_gradient2(low = "blue", mid = "grey", high = "red", space = "Lab" ) +  theme
+geom_abline(slope=modelEQ2$coefficients["log(Mass_Pantheria)"],intercept=modelEQ2$coefficients["(Intercept)"],linetype="dashed")
+geom_abline(slope=modelEQ$model$coef["log(Mass_Pantheria)"],intercept=modelEQ$model$coef["(Intercept)"]) + xlab("log(Body mass") +ylab("log(Brain mass)")
regplot
### Plot avec les familles
Family<-c(Ecology_complete_S_all$Family)
names(Family)<-c(Ecology_complete_S_all$Species)
Suborder<-c(Ecology_complete_S_all$Suborder)
names(Suborder)<-c(Ecology_complete_S_all$Species)
sp<-ggplot(Ecology_complete_S_all, aes(x= log(Mass_Pantheria),y=log(BM), color=Family, shape=Suborder)) + geom_point()
sp + geom_point(size = 4) + scale_color_manual(values = c("#000000","#FF0000","#993300", "#FF9900","#FFFF00","#66FF33","#339933","#33FFFF","#3333FF", "#9933FF","#FF3399", "#cccccc","#FFFFFF")) +
geom_abline(slope=modelEQ$model$coef["log(Mass_Pantheria)"],intercept=modelEQ$model$coef["(Intercept)"]) +  theme  + xlab("log(Body mass)") +ylab("log(Brain mass)")


## Etude du pattern of tempo of encephalization
#D'abord sur le EQ provenant de la pgls
#on extrait la colonne EQ qu'on met sous forme de vecteur et on nomme les éléments avec les noms des espèces pour ensuite matcher avec la fonction et l'arbre
EQsevVector=as.numeric(Ecology_complete_S_all$EQsev)
names(EQsevVector)=Ecology_complete_S_all$Species

#on fait un arbre qui ne contient que les mêmes espèces que le dataset1
PhyloTreePrunned=PhyloTree
for(SP in PhyloTreePrunned$tip.label)
{
  if (!(SP %in% Ecology_complete_S_all$Species))
  {
    PhyloTreePrunned=drop.tip(PhyloTreePrunned,tip=SP)
  }
}
```

```
RRres=RRphylo(tree=PhyloTreePrunned,y=EQsevVector)

#ploter l'arbre avec les noms d'espèces et numéros de nodes
#png("EQ_evolution_rates/arbre_EQ_rate.png",height=2000,width=1500)
#plot(RRres$tree,cex=1,show.tip.label=TRUE)
#nodelabels(frame="none",cex=1, col="blue")
#dev.off()

#calcule les points de changement de rate du EQ aux noeuds de l'arbre
Shift<-search.shift(RR=RRres,status.type="clade", foldername=tempdir())
Shift$all.clades
Shift$single.clades
RRres$rates

### vOIR L'ARBRE AVEC LES BON NUM2ROS DES JOEUDS
Shift$rates
plot(RRres$tree,cex=0.4)
nodelabels(frame="none",cex=0.4, col="red")

####Testing the robustness of shifts search method results to sampling effects and phylogenetic uncertainty
overfit<-overfitRR(RR=RRres,y=EQsevVector,swap.args =list(si=0.1,si2=0.1), shift.args = list(node=rownames(Shift$single.clades)),nsim=100,clus=cc)
overfit$shift.results$clade




# Données la taille relative du cerveau
trait1<-c(modelEQ$residuals)
names(trait1)<-as.character(dimnames(modelEQ$residuals)[[1]])
#On mappe ensuite les valeurs sur la phylogénie
obj<-contMap(RRres$tree,trait1)
obj<-setMap(obj,colors=c("blue","gray","red"), space="Lab")
plot(obj,lwd=7,type="fan")
plot(obj,fsize=c(0.40,0.6),outline=FALSE,lwd=c(5,8),leg.txt="Phylogenetic Encephalization Quotient", type="fan")
values<-c(RRres$rates[1:173,])
br<-brewer.pal(n = 9, name = "Greys")
gradient<-gradient.rect(-1,0.8,1,0.95,nslices=200,col=br)
nodelabels(pie=obj$ace,bg = gradient, frame = "c", cex=0.2)


#Par famille
FamilyVector=as.character(Ecology_complete_S_all$Family)
names(FamilyVector)=Ecology_complete_S_all$Species

Shift2<-search.shift(RR=RRres,status.type="sparse",state=FamilyVector,foldername=tempdir())
Shift2$state.results
```

```
## PART 2 ##

## Importe et affiche l'excel du dataset2 (BM, body mass + variables sur 124 species)
# ATTENTION: bien changer dans l'excel les points en virgules, car étant un excel français, il prenait les chiffres pour du texte
Ecology_complete_S02 <- read_excel("C:/Users/mmar3/Dropbox/Dossier pro/Projet FYSSEN/Article - Brain mass/Manuscript/V2/Script/brain/Ecology_complete_S02.xlsx")

# Mais d'abord calculer Temperature en Kelvin et rajouter une colonne au tableau (TempK)
# Aussi on cree des log de geographic_range et home_range car ces variables ont des valeurs très étandues
Ecology_complete_S02$TempK<-Ecology_complete_S02$Temperature+273.15
Ecology_complete_S02$LogGeographic_range<-log(Ecology_complete_S02$Geographic_range)
Ecology_complete_S02$LogHome_range<-log(Ecology_complete_S02$Home_range)

## REINCORPORER LE EQ CALCULé EN PART1 (CAR BASé SUR + D'ESPECES) dans le dataset2
# il vaut mieux pour la suite des analyses rester sur cette valeur de EQ calculée 1 fois

fitEQ= gls(log(BM)~log(Mass_Pantheria),data=Ecology_complete_S02)
Ecology_complete_S02$EQ<-fitEQ$residuals
Ecology_complete_S02=left_join(Ecology_complete_S02,Ecology_complete_S_all[,c("EQsev","EQsev2","Species")],on="Species")

#Ici on choisit quel EQ utiliser pour la suite (EQsev= from pgls, et EQsev2= from gls) et qui se retrouve comme valeur de EQ dans le dataset2
Ecology_complete_S02$EQ=Ecology_complete_S02$EQsev


## On fait une version des data en convertisant les variables catégorielles en factors. C'est pour "dire" à R que ces variables sont effectivement des catégories
Ecology_complete_S02bis= data.frame(Ecology_complete_S02)
VarFactor= c("Diet","Locomotion","Hibernation","Social_type","Activity")
for(V in VarFactor)
{
  Ecology_complete_S02bis[,V]<-as.factor(Ecology_complete_S02bis[,V])
}

## On cree des listes de variables explicatives à utiliser pour la suite.
AllVar=
c("Diet","Locomotion","Hibernation","Group_size","Social_type","Gestation","Interbirth","Litter_size","Weaning","LogGeographic_range","LogHome_range","TempK")
AllVarEQ_vulnerability=
c("Diet","Locomotion","Hibernation","Group_size","Social_type","Gestation","Interbirth","Litter_size","Weaning","LogGeographic_range","LogHome_range","TempK","Activity","Vulnerability","EQ")
```

```
AllVarEQ=
c("Diet","Locomotion","Hibernation","Group_size","Social_type","Gestation","Interbirth","Litter_size
","Weaning","LogGeographic_range","LogHome_range","TempK","Activity","EQ")
VarCat= c("Diet","Locomotion","Hibernation","Social_type","Activity")
VarNum=
c("Group_size","Gestation","Interbirth","Litter_size","Weaning","LogGeographic_range","LogHome_
range","TempK","Vulnerability","EQ")
AllVarEQ_Socialtype=
c("Diet","Locomotion","Hibernation","Social_type","Gestation","Interbirth","Litter_size","Weaning","
LogGeographic_range","LogHome_range","TempK","Activity","EQ")
AllVarEQ_Groupsize=
c("Diet","Locomotion","Hibernation","Group_size","Gestation","Interbirth","Litter_size","Weaning","
LogGeographic_range","LogHome_range","TempK","Activity","EQ")
AllVar_families=
c("Diet","Locomotion","Hibernation","Group_size","Social_type","Gestation","Interbirth","Litter_size
","Weaning","LogGeographic_range","LogHome_range","TempK","Family","Suborder","EQ")

## Test si les variables d'intérêt sont corrélées entre elles + plot associé
# PROBLEME: certaines variables sont des catégories. Donc pas de corrélation possible avec la
méthode de spearman seule.
# SOLUTION: methode fancy ci-dessous, pour lesquelles il faut installer les packages: tidyverse et
rcompanion.

# Calculate a pairwise association between all variables in a data-frame. In particular nominal vs
nominal with Chi-square, numeric vs numeric with Pearson correlation, and nominal vs numeric with
ANOVA.
# Adopted from https://stackoverflow.com/a/52557631/590437
mixed_assoc = function(df, cor_method="spearman", adjust_cramersv_bias=TRUE){
  df_comb = expand.grid(names(df), names(df),  stringsAsFactors = F) %>% set_names("X1", "X2")

  is_nominal = function(x) class(x) %in% c("factor", "character")
  # https://community.rstudio.com/t/why-is-purr-is-numeric-deprecated/3559
  # https://github.com/r-lib/rlang/issues/781
  is_numeric <- function(x) { is.integer(x) || is_double(x)}

  f = function(xName,yName) {
    x =  pull(df, xName)
    y =  pull(df, yName)

    result = if(is_nominal(x) && is_nominal(y)){
      # use bias corrected cramersV as described in
https://rdrr.io/cran/rcompanion/man/cramerV.html
      cv = cramerV(as.character(x), as.character(y), bias.correct = adjust_cramersv_bias)
      data.frame(xName, yName, assoc=cv, type="cramersV")

    }else if(is_numeric(x) && is_numeric(y)){
      correlation = cor(x, y, method=cor_method, use="complete.obs")
      data.frame(xName, yName, assoc=correlation, type="correlation")

    }else if(is_numeric(x) && is_nominal(y)){
      # from https://stats.stackexchange.com/questions/119835/correlation-between-a-nominal-iv-
and-a-continuous-dv-variable/124618#124618
```

```
    r_squared = summary(lm(x ~ y))$r.squared
    data.frame(xName, yName, assoc=sqrt(r_squared), type="anova")

   }else if(is_nominal(x) && is_numeric(y)){
    r_squared = summary(lm(y ~x))$r.squared
    data.frame(xName, yName, assoc=sqrt(r_squared), type="anova")

   }else {
    warning(paste("unmatched column type combination: ", class(x), class(y)))
   }

   # finally add complete obs number and ratio to table
   result %>% mutate(complete_obs_pairs=sum(!is.na(x) & !is.na(y)),
complete_obs_ratio=complete_obs_pairs/length(x)) %>% rename(x=xName, y=yName)
  }

  # apply function to each variable combination
  map2_df(df_comb$X1, df_comb$X2, f)

}
```

goes with the previous one and computes "pvalues" for associations. For spearman, it is the pvalue, for cramerV, the pvalue is derived from the chisquared test, and for the anova, from the anova itself.

```
mixed_assoc_pvals = function(df, cor_method="spearman", adjust_cramersv_bias=TRUE){
  df_comb = expand.grid(names(df), names(df),  stringsAsFactors = F) %>% set_names("X1", "X2")

  is_nominal = function(x) class(x) %in% c("factor", "character")
  # https://community.rstudio.com/t/why-is-purr-is-numeric-deprecated/3559
  # https://github.com/r-lib/rlang/issues/781
  is_numeric <- function(x) { is.integer(x) || is_double(x)}

  f = function(xName,yName) {
   x =  pull(df, xName)
   y =  pull(df, yName)

   result = if(is_nominal(x) && is_nominal(y)){
    # use bias corrected cramersV as described in
https://rdrr.io/cran/rcompanion/man/cramerV.html
    #cv = cramerV(as.character(x), as.character(y), bias.correct = adjust_cramersv_bias)
    ct = chisq.test(as.character(x), as.character(y), simulate.p.value = TRUE)
    data.frame(xName, yName, pval=ct$p.value, type="cramersV")

   }else if(is_numeric(x) && is_numeric(y)){
    correlation = cor.test(x, y, method=cor_method, use="complete.obs",exact = FALSE)
    data.frame(xName, yName, pval=correlation$p.value, type="correlation")

   }else if(is_numeric(x) && is_nominal(y)){
    # from https://stats.stackexchange.com/questions/119835/correlation-between-a-nominal-iv-
and-a-continuous-dv-variable/124618#124618
    f = summary(lm(x ~ y))$fstatistic
```

```
    pv <- unname(pf(f[1],f[2],f[3],lower.tail = F))
    data.frame(xName, yName, pval=pv, type="anova")

  }else if(is_nominal(x) && is_numeric(y)){
    #r_squared = summary(lm(y ~x))$r.squared
    f = summary(lm(y ~ x))$fstatistic
    pv <- unname(pf(f[1],f[2],f[3],lower.tail = F))
    data.frame(xName, yName, pval=pv, type="anova")

  }else {
    warning(paste("unmatched column type combination: ", class(x), class(y)))
  }

  # finally add complete obs number and ratio to table
  result %>% mutate(complete_obs_pairs=sum(!is.na(x) & !is.na(y)),
complete_obs_ratio=complete_obs_pairs/length(x)) %>% rename(x=xName, y=yName)
 }

 # apply function to each variable combination
 map2_df(df_comb$X1, df_comb$X2, f)



}


## Calcul of the correlation + inclusion of EQ in the correlation matrix
res_varcorrel= mixed_assoc(Ecology_complete_S02bis[,AllVarEQ_vulnerability])

## Calcul des pValues associées aux corrélations/associations
res_varcorrel_pvals= mixed_assoc_pvals(Ecology_complete_S02bis[,AllVarEQ_vulnerability])
#At the end, performs a benjamini Hochberg correction for multiple test
res_varcorrel_pvals$pval = p.adjust(res_varcorrel_pvals$pval, method="BH")


## Visualisation des résultats
# 1- on reformate les resultats sous forme de matrice
# 2- on plot la nouvelle matrice

m<-data.frame()
p<-data.frame()
for(V in AllVarEQ_vulnerability)
{
 for(V2 in AllVarEQ_vulnerability)
 {
  m[V,V2]<-res_varcorrel[(res_varcorrel$x==V)&(res_varcorrel$y==V2),"assoc"]
  p[V,V2]<-res_varcorrel_pvals[(res_varcorrel$x==V)&(res_varcorrel$y==V2),"pval"]

 }
}
```

```
m<-data.matrix(m)
p<-data.matrix(p)

col3 <- colorRampPalette (c("blue", "white", "red"))
corrplot(m, p.mat=p, method="circle",type="lower",sig.level = c(.001, .01, .05), pch.col =
"black",pch.cex = .9, insig = "label_sig", col = col3(200))
corrplot(m, p.mat=p, method="color",type="lower",sig.level = c(.001, .01, .05), pch.col =
"black",pch.cex = .9, insig = "label_sig", col = col3(200))
# DONC seulement Social_type et Groupe_size sont fortement corrélées


## Importer l'arbre phylo puis calculer le pagel lambda pour chaque variable d'intérêt
# Pour évaluer le signal phylo de chaque variable
# MAIS Margot dit non faut le faire sur les résidus de la pgls

PhyloTree=read.nexus(file="C:/Users/mmar3/Dropbox/Dossier pro/Projet FYSSEN/Article - Brain
mass/Manuscript/V2/Script/brain/Phy_Slater.nex")
#plot(PhyloTree,cex=.1)


#Pour les variables numériques
# (on utilise le log sauf pour EQ of course qui est déjà un log)
PhyloTab=data.frame(row.names=AllVarEQ)
for(V in VarNum)
{
  trait=Ecology_complete_S02bis[,V]
  names(trait)=Ecology_complete_S02bis$Species
  Pphyl=phylosig(PhyloTree,x=trait,method="lambda",test=TRUE,nsim=5000)
  PhyloTab[V,"lambda"]=Pphyl$lambda
  PhyloTab[V,"Pval"]=Pphyl$P
}

#Pour les variables catégorielles
for(V in VarCat)
{

trait=dummy_cols(Ecology_complete_S02bis[,c(V,"Species")],select_columns=c(V),remove_selected_
columns=TRUE)
  allPhy=numeric()
  allP=numeric()
  i=1
  for(C in colnames(trait))
  {
    if(C!="Species")
    {
      subtrait=trait[,C]
      names(subtrait)=Ecology_complete_S02bis$Species
      Pphyl=phylosig(PhyloTree,x=subtrait,method="lambda",test=TRUE,nsim=5000)
      PhyloTab[C,"lambda"]=Pphyl$lambda
      PhyloTab[C,"Pval"]=Pphyl$P
      allPhy[i]=Pphyl$lambda
      allP[i]=Pphyl$P
```

```
    i=i+1
   }

  }
  PhyloTab[V,"lambda"]=max(allPhy)
  PhyloTab[V,"Pval"]=min(allP)
}
```

```
## Exporter excel
write.csv2(PhyloTab, file="PhyloSignalperVariable.csv")
# DONC toutes les variables ont un signal phylo.Mais peut-être pourri



## Analyse de l'influence des predictors sur le EQ
# Creation d'un model predictif minimal avec les variables predicteurs
# -> PGLS (et aussi analyse sans compensation phylo pour voir les differences et mieux expliquer les resultats)
# Modele avec toutes variables, puis  model minimal
# -> Faire pour toutes espèces, puis par suborder et par famille pour voir les possiblement differents
CompData=comparative.data(PhyloTree,data=Ecology_complete_S02bis[,c(AllVarEQ,"Species")],names.col="Species")

## Maximal model, with all variables & for all species
model1=pgls(EQ~.,data=CompData,lambda="ML")
summary(model1)
#Donc l'ensemble des variables expliquent environ 25% de la variance de l'EQ.
#Mais c'est non significatif, et l'adjusted R-squarred est trop petit par rapport au multiple R-squarred, donc il y a beaucoup de variables non-informatives.


## Donc on cree des fonctions qui explorent tous les modèles possibles par ajout successif de variables, afin de selectionner le meilleur model minimal explicatif de l'EQ. En faisant bien des pgls, qui compensent le signal phylogénétique des variables.
#Pour cela on utilise le BIC, qui est une mesure servant à comparer les modèles. + le BIC est petit, plus le modèle est meilleur. C'est un trade-off entre le nombre de variables et la qualité de prédiction.

#declaration de la fonction
explore_additional_var<-function(var_to_check, previous_formula, dataset ){
```

```
  results<-data.frame()
  v="previous_model"

  res<-tryCatch(
    {
      PrevModel <- pgls(as.formula(previous_formula), data = dataset, lambda="ML")
      BIC(PrevModel)
    }, error = function(err){
      #print(paste("Error (silenced):  ",err))
      return(100000)
    }
  )

  results[v,"BIC"]<-res

  for(v in var_to_check){
    if(length(unique(dataset$data[,v]))>1) #manage the case where the variable is constant => would
throw an error
    {
      current_formula<-paste(previous_formula,v,sep=' + ')

      res<-tryCatch(
        {
          NextModelCandidate <- pgls(as.formula(current_formula), data = dataset, lambda="ML")
          BIC(NextModelCandidate)
        }, error = function(err){
          #print(paste("Error (silenced):  ",err))
          return(100000)
        }
      )
      results[v,"BIC"]<-res
      #NextModelCandidate <- pgls(as.formula(current_formula), data = dataset, lambda="ML")
      #results[v,"BIC"]<-NextModelCandidate$aic
    }
    else
    {
      results[v,"BIC"]<-100000 #something very large
    }

  }
  return(results)
}

#fait une loop et augmente progressivement jusqu'à ce que le BIC ne s'améliore plus
optimize_model<-function(var_to_check,dataset){
  #Utilisation de la fonction
  var_to_check=var_to_check[var_to_check!="EQ"]
  previous_formula="EQ ~ 1"
  #previous_model<-pgls(as.formula(previous_formula) , data = dataset, lambda="ML")
  explo_result=explore_additional_var(var_to_check, previous_formula, dataset=dataset)
  winner=rownames(explo_result)[which.min(explo_result$BIC)]
  print(paste("round",0," winner=",winner," with BIC=",min(explo_result$BIC)))
```

```
 for(i in seq(length(var_to_check))){
   if(winner!="previous_model")
   {
     var_to_check=var_to_check[var_to_check!=winner]
     previous_formula<-paste(previous_formula,winner,sep=' + ')
     explo_result=explore_additional_var(var_to_check, previous_formula, dataset=dataset)
     winner=rownames(explo_result)[which.min(explo_result$BIC)]
     print(paste("round",i," winner=",winner," with BIC=",min(explo_result$BIC)))

   }
 }
 winningModel = pgls(as.formula(previous_formula) , data = dataset, lambda="ML")
 return(winningModel)

}


### Pour le dataset "Social type"
winningModel=optimize_model(var_to_check = AllVarEQ_Socialtype, dataset=CompData)
summary(winningModel)
### Pour le dataset "Group_size"
winningModel=optimize_model(var_to_check = AllVarEQ_Groupsize, dataset=CompData)
summary(winningModel)
## Utilise les fonctions pours faire l'optimisation de modèle dans le cas général (toutes les espèces)
winningModel=optimize_model(var_to_check = AllVarEQ, dataset=CompData)
summary(winningModel)



# Donc model general = geographic_range + home_range pour expliquer envir 13% de la variance de EQ chez toutes espèces confondues. très significatif

## Utilise les fonctions pours faire l'optimisation de modèle dans le cas suborders
#Feliformia

#we need to droplevel in case some fators do not exist in sub group
### Social type
feli_data=droplevels(Ecology_complete_S02bis[Ecology_complete_S02bis$Suborder=="Feliformia",c(AllVarEQ,"Species")])
Suborder1CompData=comparative.data(PhyloTree,data=feli_data,names.col="Species")
winningModel=optimize_model(var_to_check = AllVarEQ_Socialtype, dataset=Suborder1CompData)
summary(winningModel)
### Group size
feli_data=droplevels(Ecology_complete_S02bis[Ecology_complete_S02bis$Suborder=="Feliformia",c(AllVarEQ,"Species")])
Suborder1CompData=comparative.data(PhyloTree,data=feli_data,names.col="Species")
winningModel=optimize_model(var_to_check = AllVarEQ_Groupsize, dataset=Suborder1CompData)
summary(winningModel)
### All
feli_data=droplevels(Ecology_complete_S02bis[Ecology_complete_S02bis$Suborder=="Feliformia",c(AllVarEQ,"Species")])
Suborder1CompData=comparative.data(PhyloTree,data=feli_data,names.col="Species")
```

```
winningModel=optimize_model(var_to_check = AllVarEQ, dataset=Suborder1CompData)
summary(winningModel)
#Donc ici c'est la température
### Phylo signal lambda
phylosig(PhyloTree,winningModel$residuals, method="lambda", test=TRUE,nsim=999)


#Caniformia
### Social type
cani_data=droplevels(Ecology_complete_S02bis[Ecology_complete_S02bis$Suborder=="Caniformia",
c(AllVarEQ,"Species")])
Suborder2CompData=comparative.data(PhyloTree,data=cani_data,names.col="Species")
winningModel=optimize_model(var_to_check = AllVarEQ_Socialtype, dataset=Suborder2CompData)
summary(winningModel)
### Group size
cani_data=droplevels(Ecology_complete_S02bis[Ecology_complete_S02bis$Suborder=="Caniformia",
c(AllVarEQ,"Species")])
Suborder2CompData=comparative.data(PhyloTree,data=cani_data,names.col="Species")
winningModel=optimize_model(var_to_check = AllVarEQ_Groupsize, dataset=Suborder2CompData)
summary(winningModel)
### All
cani_data=droplevels(Ecology_complete_S02bis[Ecology_complete_S02bis$Suborder=="Caniformia",
c(AllVarEQ,"Species")])
Suborder2CompData=comparative.data(PhyloTree,data=cani_data,names.col="Species")
winningModel=optimize_model(var_to_check = AllVarEQ, dataset=Suborder2CompData)
summary(winningModel)
### Phylo signal lambda
phylosig(PhyloTree,winningModel$residuals, method="lambda", test=TRUE,nsim=999)

# Ici c'est geo_range, home_range et hibernation


### Mustelidae
## SOcial type
Must_data=droplevels(Ecology_complete_S02bis[Ecology_complete_S02bis$Family=="Mustelidae",c
(AllVarEQ,"Species")])
Must_CompData=comparative.data(PhyloTree,data=Must_data,names.col="Species")
winningModel=optimize_model(var_to_check = AllVarEQ_Socialtype, dataset=Must_CompData)
summary(winningModel)
## Group size
Must_data=droplevels(Ecology_complete_S02bis[Ecology_complete_S02bis$Family=="Mustelidae",c
(AllVarEQ,"Species")])
Must_CompData=comparative.data(PhyloTree,data=Must_data,names.col="Species")
winningModel=optimize_model(var_to_check = AllVarEQ_Groupsize, dataset=Must_CompData)
summary(winningModel)


library(phytools)
?phylosig


## Utilise les fonctions pours faire l'optimisation de modèle dans le cas family
```

```
# On dit que faut minimum 3 espèces par famille
all_fam=unique(Ecology_complete_S02bis$Family)
for(f in all_fam){
  print("")
  print("")
  print("")
  print(paste("=================================="))
  print(paste("========",f,"=========="))
  print("")


fam_data=droplevels(Ecology_complete_S02bis[Ecology_complete_S02bis$Family==f,c(AllVarEQ,"Sp
ecies")])
  if(nrow(fam_data)>3){
    famCompData=comparative.data(PhyloTree,data=fam_data,names.col="Species")
    winningModel=optimize_model(var_to_check = AllVarEQ, dataset=famCompData)
    print(summary(winningModel))
  }
  else{
    print("Not enough species in this family to build models")
  }


}



## Plots des EQ VS chaque variables predictor avec code couleur par famille et code étoile/carré par
suborder
VarCat= c("Diet","Locomotion","Hibernation","Social_type","Activity")
VarNum2=
c("Group_size","Gestation","Interbirth","Litter_size","Weaning","LogGeographic_range","LogHome_
range","TempK")



save_fig<-function(fig_obj, filename){
  save_path="C:/Users/mmar3/Dropbox/Dossier pro/Projet FYSSEN/Article - Brain
mass/Manuscript/V2/Script/brain/Figures"
  file_name = paste(save_path,filename,".svg",sep="")
  postscript(file = file_name,width=10,height=7)
  print(fig_obj)
  dev.off()
  file_name = paste(save_path,filename,".png",sep="")
  png(file = file_name,width=800,height=600)
  print(fig_obj)
  dev.off()
}

i=1
pp=list()
for (p in VarCat)
```

```
{
  pp[[i]]<-
ggplot(data=Ecology_complete_S02bis,aes_string(x=p,y="EQ"))+geom_violin(trim=FALSE)+theme+ge
om_jitter(position=position_jitter(0.07),size=2,aes(color=Family,shape=Suborder))
  save_fig(pp[[i]],paste("EQ_vs_",p))
  i=i+1
}

for (p in VarNum2)
{
  pp[[i]]<-
ggplot(data=Ecology_complete_S02bis,aes_string(x=p,y="EQ"))+theme+geom_point(size=2,aes(color
=Family,shape=Suborder))
  save_fig(pp[[i]],paste("EQ_vs_",p))
  i=i+1
}

print(pp[[2]])
```

```
############################################## ## Test between vulnerability and EQ

### Carnivora entier order
CompData2=comparative.data(PhyloTree,data=Ecology_complete_S02bis[,c(AllVarEQ_vulnerability,"
Species")],names.col="Species")
model_v=pgls(Ecology_complete_S02bis$EQsev~Ecology_complete_S02bis$Vulnerability,data=Comp
Data2,lambda="ML")
summary(model_v)
sp<-ggplot(Ecology_complete_S02bis, aes(x= Vulnerability,y=EQsev, color=Family, shape=Suborder))
+ geom_point()
sp + geom_point(size = 4) + scale_color_manual(values = c("#000000","#FF0000","#993300",
"#FF9900","#FFFF00","#66FF33","#339933","#33FFFF","#3333FF", "#9933FF","#FF3399",
"#cccccc","#FFFFFF"))  +  theme  + xlab("EQ") +ylab("log(Brain mass)")


### Caniformia suborder
Canif_data=droplevels(Ecology_complete_S02bis[Ecology_complete_S02bis$Suborder=="Caniformia
",c(AllVarEQ_vulnerability,"Species")])
Canif_CompData2=comparative.data(PhyloTree,data=Canif_data[,c(AllVarEQ_vulnerability,"Species")
],names.col="Species")
Canif_model_v=pgls(Canif_data$EQ~Canif_data$Vulnerability,data=Canif_CompData2,lambda="ML"
)
summary(Canif_model_v)
sp<-ggplot(Canif_data, aes(x= Canif_data$Vulnerability,y=Canif_data$EQ)) + geom_point()
sp + geom_point(size = 4)   +  theme  + xlab("EQ") +ylab("log(Brain mass)")

### Feliformia suborder
```

```
Felif_data=droplevels(Ecology_complete_S02bis[Ecology_complete_S02bis$Suborder=="Feliformia",
c(AllVarEQ_vulnerability,"Species")])
Felif_CompData2=comparative.data(PhyloTree,data=Felif_data[,c(AllVarEQ_vulnerability,"Species")],
names.col="Species")
Felif_model_v=pgls(Felif_data$EQ~Felif_data$Vulnerability,data=Felif_CompData2,lambda="ML")
summary(Felif_model_v)
sp<-ggplot(Felif_data, aes(x= Felif_data$Vulnerability,y=Felif_data$EQ)) + geom_point()
sp + geom_point(size = 4)   +  theme  + xlab("EQ") +ylab("log(Brain mass)")

### Canidae family
Canid_data=droplevels(Ecology_complete_S02bis[Ecology_complete_S02bis$Family=="Canidae",c(Al
lVarEQ_vulnerability,"Species")])
Canid_CompData2=comparative.data(PhyloTree,data=Canid_data[,c(AllVarEQ_vulnerability,"Species
")],names.col="Species")
Canid_model_v=pgls(Canid_data$EQ~Canid_data$Vulnerability,data=Canid_CompData2,lambda="M
L")
summary(Canid_model_v)
sp<-ggplot(Felif_data, aes(x= Canid_data$Vulnerability,y=Canid_data$EQ)) + geom_point()
sp + geom_point(size = 4)   +  theme  + xlab("EQ") +ylab("log(Brain mass)")

### Felidae family
Felid_data=droplevels(Ecology_complete_S02bis[Ecology_complete_S02bis$Family=="Felidae",c(AllV
arEQ_vulnerability,"Species")])
Felid_CompData2=comparative.data(PhyloTree,data=Felid_data[,c(AllVarEQ_vulnerability,"Species")]
,names.col="Species")
Felid_model_v=pgls(Felid_data$EQ~Felid_data$Vulnerability,data=Felid_CompData2,lambda="ML")
summary(Felid_model_v)
sp<-ggplot(Felif_data, aes(x= Felid_data$Vulnerability,y=Felid_data$EQ)) + geom_point()
sp + geom_point(size = 4)   +  theme  + xlab("EQ") +ylab("log(Brain mass)")

### Mustelidae family
Must_data=droplevels(Ecology_complete_S02bis[Ecology_complete_S02bis$Family=="Mustelidae",c
(AllVarEQ_vulnerability,"Species")])
Must_CompData2=comparative.data(PhyloTree,data=Must_data[,c(AllVarEQ_vulnerability,"Species"
)],names.col="Species")
Must_model_v=pgls(Must_data$EQ~Must_data$Vulnerability,data=Must_CompData2,lambda="ML"
)
summary(Must_model_v)
sp<-ggplot(Must_data, aes(x= Must_data$Vulnerability,y=Must_data$EQ)) + geom_point()
sp + geom_point(size = 4)   +  theme  + xlab("EQ") +ylab("log(Brain mass)")




##################################### Plot par famille/ sous ordre
p <- ggplot(Ecology_complete_S_all, aes(x=Family, y=EQsev)) + geom_boxplot()
p + geom_jitter(shape=16, size=3, position=position_jitter(0.1))  +  theme  + xlab("Encephalization
quotient") +ylab("Family") + geom_boxplot(fill=c("#000000","#FF0000","#993300",
"#FF9900","#FFFF00","#66FF33","#339933","#33FFFF","#3333FF", "#9933FF","#FF3399",
"#cccccc","#FFFFFF"), color="black")
```

```
p <- ggplot(Ecology_complete_S_all, aes(x=Suborder, y=EQsev)) + geom_boxplot()
p + geom_jitter(shape=16, size=3, position=position_jitter(0.1))  +  theme  + xlab("Encephalization quotient") +ylab("Sub-order")


##### Goegraphic range
sp<-ggplot(Ecology_complete_S02bis, aes(x= log(Geographic_range),y=EQsev, color=Family, shape=Suborder)) + geom_point()
sp + geom_point(size = 4) + scale_color_manual(values = c("#000000","#FF0000","#993300", "#FF9900","#FFFF00","#66FF33","#339933","#33FFFF","#3333FF", "#9933FF","#FF3399", "#cccccc","#FFFFFF")) + theme  + xlab("log(Geographic range)") +ylab("Encephalization Quotient")



c("#000000","#FF0000","#993300", 
"#FF9900","#FFFF00","#66FF33","#339933","#33FFFF","#3333FF", "#9933FF","#FF3399", 
"#cccccc","#FFFFFF")

##################################### Arbre de décision sans les familles
all_data = Ecology_complete_S02bis[,AllVarEQ]
# To tune & assess decision trees performance, we need to keep some of the data out of the tree generation
set.seed(42)#comment this out to have different random splits
split_data=initial_split(all_data,prop = 0.7) #70% train, 30% test
train_data=training(split_data)
test_data=testing(split_data)

## computes the tree based on the train data
d_model=rpart(EQ~., data=train_data, method="anova")
summary(d_model)

###### Compute the prediction capability
compute_rsquared=function(pred,y,is_testset){
  #based on https://stackoverflow.com/questions/25691127/r-squared-on-test-data/36727900
  SS.total      <- sum((y - mean(y))^2)
  SS.residual   <- sum((y - pred)^2)
  SS.regression <- sum((pred - mean(y))^2)
  if(is_testset){
    return(SS.regression/SS.total)
  }
  else{
    return(1 - SS.residual/SS.total )

  }
}

compute_rmse=function(pred,y){
  return(sqrt(mean((pred-y)^2)))
```

```
}

# Compute performance on test dataset
test.pred <- predict(d_model, newdata = test_data)
test.y    <- test_data$EQ
pred_rsquared=compute_rsquared(pred=test.pred,y=test.y,is_testset=TRUE)
pred_rmse=compute_rmse(pred=test.pred,y=test.y)
print(paste("test set performance: r-squared=",pred_rsquared," RMSE=",pred_rmse))

# Compute performance on train dataset
train.pred <- predict(d_model, newdata = train_data)
train.y    <- train_data$EQ
train_rsquared=compute_rsquared(pred=train.pred,y=train.y,is_testset=TRUE)
train_rmse=compute_rmse(pred=train.pred,y=train.y)
print(paste("train set performance: r-squared=",train_rsquared," RMSE=",train_rmse))

#### represent which Species are where on the tree
leaf = rpart.predict.leaves(d_model,newdata=Ecology_complete_S02bis, type="where")
annotated_data=Ecology_complete_S02bis
annotated_data$leaf=leaf
family_per_node = annotated_data %>% count(leaf,Family)
species_per_node = annotated_data %>% count(leaf,Species)

annotations=c()
nnodes=nrow(d_model$frame)
for(n in 1:nnodes){

  annotations=c(annotations,paste(c("
\n",species_per_node[species_per_node$leaf==n,"Species"]),sep="",collapse = '\n'))

}

#rpart.plot(d_model,  suffix = "\nfraction")
rpart.plot(d_model,  suffix = annotations)

##### represent which Families are where on the tree
leaf = rpart.predict.leaves(d_model,newdata=Ecology_complete_S02bis, type="where")
annotated_data=Ecology_complete_S02bis
annotated_data$leaf=leaf
family_per_node = annotated_data %>% count(leaf,Family)
family_per_node$famcount <- apply( family_per_node[ , c("n","Family") ] , 1 , paste , collapse = "-" )
species_per_node = annotated_data %>% count(leaf,Species)

annotations=c()
nnodes=nrow(d_model$frame)
for(n in 1:nnodes){

  annotations=c(annotations,paste(c("
\n",family_per_node[family_per_node$leaf==n,"famcount"]),sep="",collapse = '\n'))

}
```

```
#rpart.plot(d_model,  suffix = "\nfraction")
rpart.plot(d_model,  suffix = annotations)
```

```
###################################### Arbre de décision avec les familles
all_data = Ecology_complete_S02bis[,AllVar_families]
# To tune & assess decision trees performance, we need to keep some of the data out of the tree
generation
set.seed(42)#comment this out to have different random splits
split_data=initial_split(all_data,prop = 0.7) #70% train, 30% test
train_data=training(split_data)
test_data=testing(split_data)



## computes the tree based on the train data
d_model1=rpart(EQ~., data=train_data, method="anova")
summary(d_model1)

###### Compute the prediction capability
compute_rsquared=function(pred,y,is_testset){
  #based on https://stackoverflow.com/questions/25691127/r-squared-on-test-data/36727900
  SS.total      <- sum((y - mean(y))^2)
  SS.residual   <- sum((y - pred)^2)
  SS.regression <- sum((pred - mean(y))^2)
  if(is_testset){
    return(SS.regression/SS.total)
  }
  else{
    return(1 - SS.residual/SS.total )

  }
}

compute_rmse=function(pred,y){
  return(sqrt(mean((pred-y)^2)))
}

# Compute performance on test dataset
test.pred <- predict(d_model1, newdata = test_data)
test.y    <- test_data$EQ
pred_rsquared=compute_rsquared(pred=test.pred,y=test.y,is_testset=TRUE)
pred_rmse=compute_rmse(pred=test.pred,y=test.y)
print(paste("test set performance: r-squared=",pred_rsquared," RMSE=",pred_rmse))

# Compute performance on train dataset
train.pred <- predict(d_model1, newdata = train_data)
train.y    <- train_data$EQ
```

```
train_rsquared=compute_rsquared(pred=train.pred,y=train.y,is_testset=TRUE)
train_rmse=compute_rmse(pred=train.pred,y=train.y)
print(paste("train set performance: r-squared=",train_rsquared," RMSE=",train_rmse))

#### represent which Species are where on the tree
leaf = rpart.predict.leaves(d_model1,newdata=Ecology_complete_S02bis, type="where")
annotated_data=Ecology_complete_S02bis
annotated_data$leaf=leaf
family_per_node = annotated_data %>% count(leaf,Family)
species_per_node = annotated_data %>% count(leaf,Species)

annotations=c()
nnodes=nrow(d_model1$frame)
for(n in 1:nnodes){

  annotations=c(annotations,paste(c("
\n",species_per_node[species_per_node$leaf==n,"Species"]),sep="",collapse = '\n'))

}

#rpart.plot(d_model1,  suffix = "\nfraction")
rpart.plot(d_model1,  suffix = annotations)


##### represent which Families are where on the tree
leaf = rpart.predict.leaves(d_model1,newdata=Ecology_complete_S02bis, type="where")
annotated_data=Ecology_complete_S02bis
annotated_data$leaf=leaf
family_per_node = annotated_data %>% count(leaf,Family)
family_per_node$famcount <- apply( family_per_node[ , c("n","Family") ] , 1 , paste , collapse = "-" )
species_per_node = annotated_data %>% count(leaf,Species)

annotations=c()
nnodes=nrow(d_model1$frame)
for(n in 1:nnodes){

  annotations=c(annotations,paste(c("
\n",family_per_node[family_per_node$leaf==n,"famcount"]),sep="",collapse = '\n'))

}

#rpart.plot(d_model1,  suffix = "\nfraction")
rpart.plot(d_model1,  suffix = annotations)




############ Differences entres sous ordres
```

```
aov=aov(modelEQ$residuals~ Family, data= Ecology_complete_S_all)
summary(aov)
aov

dev.off()
TukeyHSD(aov)
boxplot(modelEQ$residuals~ Suborder)



##### EQ mean by group
mean(Ecology_complete_S_all$EQsev[which(Ecology_complete_S_all$Suborder=="Feliformia")])
length(Ecology_complete_S_all$EQsev[which(Ecology_complete_S_all$Suborder=="Feliformia")])




############ Test des différentes courbes brain size-Body mass
### Courbe pour l'ensemble des familles
asm<-sma(log(Ecology_complete_S_all$BM)~log(Ecology_complete_S_all$Mass_Pantheria),
angle.type = "deg", iter=999)
summary(asm)

### Familles
asm<-
sma(log(Ecology_complete_S_all$BM)~log(Ecology_complete_S_all$Mass_Pantheria)*Ecology_comp
lete_S_all$Family, angle.type = "deg", iter=999)
summary(asm)
str(asm)
cb1 <- c("#FF0000","#993300", "#FF9900","#FFFF00","#66FF33","#339933","#33FFFF", "#FF3399",
        "#cccccc","#FFFFFF")
plot(asm, cex=0.7, pch=16, col=cb1)
asm<-
sma(log(Ecology_complete_S_all$BM)~log(Ecology_complete_S_all$Mass_Pantheria)*Ecology_comp
lete_S_all$Family, angle.type = "deg", iter=999)
summary(asm)

### Sous-ordres
asm<-
sma(log(Ecology_complete_S_all$BM)~log(Ecology_complete_S_all$Mass_Pantheria)*Ecology_comp
lete_S_all$Suborder, angle.type = "deg", iter=999)
summary(asm)
str(asm)
cb1 <- c("#66FF33","#FF3399")
plot(asm, cex=0.7, pch=16, col=cb1)
asm<-
sma(log(Ecology_complete_S_all$BM)~log(Ecology_complete_S_all$Mass_Pantheria)*Ecology_comp
lete_S_all$Suborder, angle.type = "deg", iter=999)
summary(asm)
```

```
############ Test for a correlation between divergence time and EQ

n<-length(PhyloTreePrunned$tip.label)
ee<-setNames(PhyloTreePrunned$edge.length[sapply(1:n,function(x,y)
which(y==x),y=PhyloTreePrunned$edge[,2])],PhyloTreePrunned$tip.label)

EQ_num<-as.numeric(Ecology_complete_S_all$EQsev)
names(EQ_num)<-dimnames(Ecology_complete_S_all$EQsev)[[1]]
bind<-cbind(EQ_num,ee,GR)

setwd("C:/Users/mmar3/Dropbox/Dossier pro/Projet FYSSEN/Article - Brain mass/Manuscript/V11 -
2em soumission (Communications Biology)")
write.csv2(bind, file="TEST.csv")

Data <- read_excel("C:/Users/mmar3/Dropbox/Dossier pro/Projet FYSSEN/Article - Brain
mass/Manuscript/V11 - 2em soumission (Communications Biology)/Age.xlsx")
### Test with 174 sp between agge and EQ
library("ggpubr")
ggscatter(Data, x = "EQ_num", y = "ee",
        add = "reg.line", conf.int = TRUE,
        cor.coef = TRUE, cor.method = "pearson", label="Species")


#### Test with 124 sp
Data2 <- read_excel("C:/Users/mmar3/Dropbox/Dossier pro/Projet FYSSEN/Article - Brain
mass/Manuscript/V11 - 2em soumission (Communications Biology)/Age_GR.xlsx")
Data2$GR<-as.numeric(Data2$GR)
log_GR<-log(Data2$GR)
matrix<-cbind(Data2,log_GR)

ggscatter(Data2, x = "EQ_num", y = "ee",
        add = "reg.line", conf.int = TRUE,
        cor.coef = TRUE, cor.method = "pearson", label="Species")



ggscatter(matrix, x = "log_GR", y = "ee",
        add = "reg.line", conf.int = TRUE,
        cor.coef = TRUE, cor.method = "pearson", label="Species")



##### Same but with a PGLS analyses
```

```
# Uploading phylogeny
setwd("C:/Users/mmar3/Dropbox/Dossier pro/Projet FYSSEN/Article - Brain mass")
phy4<-read.nexus(file="Phy_Slater.nex")
species<-list(phy4$tip.label)
speciesok<-matrix$Species
prunned.tree_S<-drop.tip(phy4,phy4$tip.label[-match(speciesok,phy4$tip.label)])
str(prunned.tree_S)
write.tree(prunned.tree_S)
is.rooted(prunned.tree_S)
is.binary.tree(prunned.tree_S)
prunned.tree_S$tip.label
str(prunned.tree_S)



comparative_data<- comparative.data(prunned.tree_S, matrix, Species, vcv=TRUE, vcv.dim=3)
PGLS<-pgls(EQ_num~ee*log_GR, data=comparative_data, lambda="ML")
anova(PGLS)
summary(PGLS)
plot(PGLS)
residuals<-PGLS$residuals
lm.lk<-pgls.profile(PGLS, which="lambda")
plot(lm.lk)





###### PGLS between DR and EQ

Data <- read_excel("C:/Users/mmar3/Dropbox/Dossier pro/Projet FYSSEN/Article - Brain
mass/Manuscript/V11 - 2em soumission (Communications Biology)/DR.xlsx")
EQ<-as.numeric(Data$EQ)
names(EQ)<-Data$Species
DR<-as.numeric(Data$DR)
names(DR)<-Data$Species

Ecology<-data.frame(Species, EQ,DR, check.rows = TRUE,fix.empty.names = TRUE)
data<-data.frame(Ecology,Species)
row.names(data)<-Species

my_data<-comparative.data(PhyloTreePrunned,Ecology,Species)
PGLS<-pgls(DR~EQ, data=my_data, lambda="ML")
anova(PGLS)
summary(PGLS)
plot(PGLS)
residuals<-PGLS$residuals
lm.lk<-pgls.profile(PGLS, which="lambda")
plot(lm.lk)
```

```
###### Test with the GR
Data2 <- read_excel("C:/Users/mmar3/Dropbox/Dossier pro/Projet FYSSEN/Article - Brain mass/Manuscript/V11 - 2em soumission (Communications Biology)/Age_GR.xlsx")
EQ<-as.numeric(Data2$EQ_num)
names(EQ)<-Data2$Species
DR<-as.numeric(Data2$DR)
names(DR)<-Data2$Species
GR<-c(Data2$GR)
GR<-as.numeric(c(GR))
GR<-log(GR)
names(GR)<-c(Data2$Species)
Species<-c(Data2$Species)
names(Species)<-c(Data2$Species)

Ecology<-data.frame(Species, EQ,DR,GR, check.rows = TRUE,fix.empty.names = TRUE)
data<-data.frame(Ecology,Species)
row.names(data)<-Species

dat.dr <- na.omit(Data)
dat.dr$animal <- dat.dr$Species
tree <- drop.tip(PhyloTree,tip=PhyloTree$tip.label[!PhyloTree$tip.label %in% dat.dr$animal])

my_data<-comparative.data(tree,Ecology,Species)
PGLS<-pgls(DR~EQ*GR, data=my_data, lambda="ML")
anova(PGLS)
summary(PGLS)
plot(PGLS)
residuals<-PGLS$residuals
lm.lk<-pgls.profile(PGLS, which="lambda")
plot(lm.lk)



######################################################################## DIET

# Carnivora
Carni_diet<-Ecology_complete_S02bis$Diet
Carni_diet<-table(Ecology_complete_S02bis$Diet)

# Caniformia
Canif_data=droplevels(Ecology_complete_S02bis[Ecology_complete_S02bis$Suborder=="Caniformia",c(AllVarEQ_vulnerability,"Species")])
Canif_CompData2=comparative.data(PhyloTree,data=Canif_data[,c(AllVarEQ_vulnerability,"Species")],names.col="Species")
Caniformia_Diet<-Canif_CompData2$data$Diet
Caniformia_Diet<-table(Canif_CompData2$data$Diet)
# Feliformia
Felif_data=droplevels(Ecology_complete_S02bis[Ecology_complete_S02bis$Suborder=="Feliformia", c(AllVarEQ_vulnerability,"Species")])
Felif_CompData2=comparative.data(PhyloTree,data=Felif_data[,c(AllVarEQ_vulnerability,"Species")], names.col="Species")
Feliformia_Diet<-Felif_CompData2$data$Diet
```

```
Feliformia_Diet<-table(Felif_CompData2$data$Diet)
# Canidae
Canid_data=droplevels(Ecology_complete_S02bis[Ecology_complete_S02bis$Family=="Canidae",c(AllVarEQ_vulnerability,"Species")])
Canid_CompData2=comparative.data(PhyloTreePrunned,data=Canid_data[,c(AllVarEQ_vulnerability,"Species")],names.col="Species")
Canidae_Diet<-Canid_CompData2$data$Diet
Canidae_Diet<-table(Canid_CompData2$data$Diet)
# Felidae
Felid_data=droplevels(Ecology_complete_S02bis[Ecology_complete_S02bis$Family=="Felidae",c(AllVarEQ_vulnerability,"Species")])
Felid_CompData2=comparative.data(PhyloTree,data=Felid_data[,c(AllVarEQ_vulnerability,"Species")],names.col="Species")
Felidae_Diet<-Felid_CompData2$data$Diet
Felidae_Diet<-table(Felid_CompData2$data$Diet)
#Mustelidae
Must_data=droplevels(Ecology_complete_S02bis[Ecology_complete_S02bis$Family=="Mustelidae",c(AllVarEQ_vulnerability,"Species")])
Must_CompData2=comparative.data(PhyloTree,data=Must_data[,c(AllVarEQ_vulnerability,"Species")],names.col="Species")
Mustelidae_Diet<-Must_CompData2$data$Diet
Mustelidae_Diet<-table(Must_CompData2$data$Diet)


#### Avec tous les taxa
Diet <- read_excel("C:/Users/mmar3/Dropbox/Dossier pro/Projet FYSSEN/Article - Brain mass/Matrix/Var_diet.xlsx")
N <- as.table(cbind(c(Diet$Carn), c(Diet$Fru),c(Diet$Herb),c(Diet$Insc),c(Diet$Omn),c(Diet$Pisc)))
dimnames(N) <- list(Taxa = c(Diet$Taxa),  diet = c("Carn","Fru","Herb","Insc","Omn","Pisc"))
P<-N/rowSums(N)
P
chisq<-chisq.test(N)
chisq
chisq$residuals
library(chisq.posthoc.test)
chisq.posthoc.test(N)
library(graphics)
mosaicplot(N, shade = TRUE, las=1,main = "Diet proportion")
?mosaicplot

#### Avec les sous ordres
chisq<-chisq.test(N[2:3,])
chisq
chisq$residuals
library(chisq.posthoc.test)
chisq.posthoc.test(N[2:3,])

#### Avec les familles
### Besoin de refaire une nouvelle table excel car des valeurs à 0 (frugivores et herbivores)
Diet <- read_excel("C:/Users/mmar3/Dropbox/Dossier pro/Projet FYSSEN/Article - Brain mass/Matrix/Var_diet2.xlsx")
N <- as.table(cbind(c(Diet$Carn),c(Diet$Insc),c(Diet$Omn),c(Diet$Pisc)))
```

```
dimnames(N) <- list(Taxa = c(Diet$Taxa),  diet = c("Carn","Insc","Omn","Pisc"))
P<-N/rowSums(N)
chisq<-chisq.test(N)
chisq
chisq$residuals
library(chisq.posthoc.test)
chisq.posthoc.test(N)

library(graphics)
mosaicplot(N, shade = TRUE, las=1,main = "Diet proportion")

?chisq.test


###################################################################### LOCOMOTION

# Carnivora
Carni_Locomotion<-Ecology_complete_S02bis$Locomotion
Carni_Locomotion<-table(Ecology_complete_S02bis$Locomotion)

# Caniformia
Canif_data=droplevels(Ecology_complete_S02bis[Ecology_complete_S02bis$Suborder=="Caniformia
",c(AllVarEQ_vulnerability,"Species")])
Canif_CompData2=comparative.data(PhyloTree,data=Canif_data[,c(AllVarEQ_vulnerability,"Species")
],names.col="Species")
Caniformia_Locomotion<-Canif_CompData2$data$Locomotion
Caniformia_Locomotion<-table(Canif_CompData2$data$Locomotion)
# Feliformia
Felif_data=droplevels(Ecology_complete_S02bis[Ecology_complete_S02bis$Suborder=="Feliformia",
c(AllVarEQ_vulnerability,"Species")])
Felif_CompData2=comparative.data(PhyloTree,data=Felif_data[,c(AllVarEQ_vulnerability,"Species")],
names.col="Species")
Feliformia_Locomotion<-Felif_CompData2$data$Locomotion
Feliformia_Locomotion<-table(Felif_CompData2$data$Locomotion)
# Canidae
Canid_data=droplevels(Ecology_complete_S02bis[Ecology_complete_S02bis$Family=="Canidae",c(Al
lVarEQ_vulnerability,"Species")])
Canid_CompData2=comparative.data(PhyloTreePrunned,data=Canid_data[,c(AllVarEQ_vulnerability,
"Species")],names.col="Species")
Canidae_Locomotion<-Canid_CompData2$data$Locomotion
Canidae_Locomotion<-table(Canid_CompData2$data$Locomotion)
# Felidae
Felid_data=droplevels(Ecology_complete_S02bis[Ecology_complete_S02bis$Family=="Felidae",c(AllV
arEQ_vulnerability,"Species")])
Felid_CompData2=comparative.data(PhyloTree,data=Felid_data[,c(AllVarEQ_vulnerability,"Species")]
,names.col="Species")
Felidae_Locomotion<-Felid_CompData2$data$Locomotion
Felidae_Locomotion<-table(Felid_CompData2$data$Locomotion)
#Mustelidae
Must_data=droplevels(Ecology_complete_S02bis[Ecology_complete_S02bis$Family=="Mustelidae",c
(AllVarEQ_vulnerability,"Species")])
```

```
Must_CompData2=comparative.data(PhyloTree,data=Must_data[,c(AllVarEQ_vulnerability,"Species"
)],names.col="Species")
Mustelidae_Locomotion<-Must_CompData2$data$Locomotion
Mustelidae_Locomotion<-table(Must_CompData2$data$Locomotion)


#### Avec tous les taxa
Locomotion <- read_excel("C:/Users/mmar3/Dropbox/Dossier pro/Projet FYSSEN/Article - Brain
mass/Matrix/Var_Locomotion.xlsx")
N <- as.table(cbind(c(Locomotion$Aq),
c(Locomotion$Arb),c(Locomotion$Sarb),c(Locomotion$Sfos),c(Locomotion$Terr)))
dimnames(N) <- list(Taxa = c(Locomotion$Taxa),  Locomotion = c("Aq","Arb","Sarb","Sfos","Terr"))
P<-N/rowSums(N)
P

chisq<-chisq.test(N)
chisq
chisq$residuals
library(chisq.posthoc.test)
chisq.posthoc.test(N)
library(graphics)
mosaicplot(N, shade = TRUE, las=1,main = "Diet proportion")


#### Avec les sous ordres
chisq<-chisq.test(N[2:3,])
chisq
chisq$residuals
library(chisq.posthoc.test)
chisq.posthoc.test(N[2:3,])

#### Avec les familles
chisq<-chisq.test(N[4:6,])
chisq
chisq$residuals
library(chisq.posthoc.test)
chisq.posthoc.test(N[2:3,])

library(graphics)
mosaicplot(N, shade = TRUE, las=1,main = "Locomotion proportion")




########################################################################  HIBERNATION

# Carnivora
Carni_Hibernation<-Ecology_complete_S02bis$Hibernation
Carni_Hibernation<-table(Ecology_complete_S02bis$Hibernation)

# Caniformia
```

```
Canif_data=droplevels(Ecology_complete_S02bis[Ecology_complete_S02bis$Suborder=="Caniformia
",c(AllVarEQ_vulnerability,"Species")])
Canif_CompData2=comparative.data(PhyloTree,data=Canif_data[,c(AllVarEQ_vulnerability,"Species")
],names.col="Species")
Caniformia_Hibernation<-Canif_CompData2$data$Hibernation
Caniformia_Hibernation<-table(Canif_CompData2$data$Hibernation)
# Feliformia
Felif_data=droplevels(Ecology_complete_S02bis[Ecology_complete_S02bis$Suborder=="Feliformia",
c(AllVarEQ_vulnerability,"Species")])
Felif_CompData2=comparative.data(PhyloTree,data=Felif_data[,c(AllVarEQ_vulnerability,"Species")],
names.col="Species")
Feliformia_Hibernation<-Felif_CompData2$data$Hibernation
Feliformia_Hibernation<-table(Felif_CompData2$data$Hibernation)
# Canidae
Canid_data=droplevels(Ecology_complete_S02bis[Ecology_complete_S02bis$Family=="Canidae",c(Al
lVarEQ_vulnerability,"Species")])
Canid_CompData2=comparative.data(PhyloTreePrunned,data=Canid_data[,c(AllVarEQ_vulnerability,
"Species")],names.col="Species")
Canidae_Hibernation<-Canid_CompData2$data$Hibernation
Canidae_Hibernation<-table(Canid_CompData2$data$Hibernation)
# Felidae
Felid_data=droplevels(Ecology_complete_S02bis[Ecology_complete_S02bis$Family=="Felidae",c(AllV
arEQ_vulnerability,"Species")])
Felid_CompData2=comparative.data(PhyloTree,data=Felid_data[,c(AllVarEQ_vulnerability,"Species")]
,names.col="Species")
Felidae_Hibernation<-Felid_CompData2$data$Hibernation
Felidae_Hibernation<-table(Felid_CompData2$data$Hibernation)
#Mustelidae
Must_data=droplevels(Ecology_complete_S02bis[Ecology_complete_S02bis$Family=="Mustelidae",c
(AllVarEQ_vulnerability,"Species")])
Must_CompData2=comparative.data(PhyloTree,data=Must_data[,c(AllVarEQ_vulnerability,"Species"
)],names.col="Species")
Mustelidae_Hibernation<-Must_CompData2$data$Hibernation
Mustelidae_Hibernation<-table(Must_CompData2$data$Hibernation)


#### Avec tous les taxa
Hibernation <- read_excel("C:/Users/mmar3/Dropbox/Dossier pro/Projet FYSSEN/Article - Brain
mass/Matrix/Var_Hibernation.xlsx")
N <- as.table(cbind(c(Hibernation$No), c(Hibernation$Hibernation)))
dimnames(N) <- list(Taxa = c(Hibernation$Taxa),  Hibernation = c("No","Hibernation"))
P<-N/rowSums(N)
P

chisq<-chisq.test(N)
chisq
chisq$residuals
library(chisq.posthoc.test)
chisq.posthoc.test(N)
library(graphics)
mosaicplot(N, shade = TRUE, las=1,main = "Hibernation proportion")
```

```
#### Avec les sous ordres
chisq<-chisq.test(N[2:3,])
chisq
chisq$residuals
library(chisq.posthoc.test)
chisq.posthoc.test(N[2:3,])

#### Avec les familles
chisq<-chisq.test(N[4:6,])
chisq
chisq$residuals
library(chisq.posthoc.test)
chisq.posthoc.test(N[2:3,])

library(graphics)
mosaicplot(N, shade = TRUE, las=1,main = "Hibernation proportion")



############################################################################ ACTIVITY

# Carnivora
Carni_Activity<-Ecology_complete_S02bis$Activity
Carni_Activity<-table(Ecology_complete_S02bis$Activity)

# Caniformia
Canif_data=droplevels(Ecology_complete_S02bis[Ecology_complete_S02bis$Suborder=="Caniformia
",c(AllVarEQ_vulnerability,"Species")])
Canif_CompData2=comparative.data(PhyloTree,data=Canif_data[,c(AllVarEQ_vulnerability,"Species")
],names.col="Species")
Caniformia_Activity<-Canif_CompData2$data$Activity
Caniformia_Activity<-table(Canif_CompData2$data$Activity)
# Feliformia
Felif_data=droplevels(Ecology_complete_S02bis[Ecology_complete_S02bis$Suborder=="Feliformia",
c(AllVarEQ_vulnerability,"Species")])
Felif_CompData2=comparative.data(PhyloTree,data=Felif_data[,c(AllVarEQ_vulnerability,"Species")],
names.col="Species")
Feliformia_Activity<-Felif_CompData2$data$Activity
Feliformia_Activity<-table(Felif_CompData2$data$Activity)
# Canidae
Canid_data=droplevels(Ecology_complete_S02bis[Ecology_complete_S02bis$Family=="Canidae",c(Al
lVarEQ_vulnerability,"Species")])
Canid_CompData2=comparative.data(PhyloTreePrunned,data=Canid_data[,c(AllVarEQ_vulnerability,
"Species")],names.col="Species")
Canidae_Activity<-Canid_CompData2$data$Activity
Canidae_Activity<-table(Canid_CompData2$data$Activity)
# Felidae
Felid_data=droplevels(Ecology_complete_S02bis[Ecology_complete_S02bis$Family=="Felidae",c(AllV
arEQ_vulnerability,"Species")])
```

```
Felid_CompData2=comparative.data(PhyloTree,data=Felid_data[,c(AllVarEQ_vulnerability,"Species")]
,names.col="Species")
Felidae_Activity<-Felid_CompData2$data$Activity
Felidae_Activity<-table(Felid_CompData2$data$Activity)
#Mustelidae
Must_data=droplevels(Ecology_complete_S02bis[Ecology_complete_S02bis$Family=="Mustelidae",c
(AllVarEQ_vulnerability,"Species")])
Must_CompData2=comparative.data(PhyloTree,data=Must_data[,c(AllVarEQ_vulnerability,"Species"
)],names.col="Species")
Mustelidae_Activity<-Must_CompData2$data$Activity
Mustelidae_Activity<-table(Must_CompData2$data$Activity)


#### Avec tous les taxa
Activity <- read_excel("C:/Users/mmar3/Dropbox/Dossier pro/Projet FYSSEN/Article - Brain
mass/Matrix/Var_Activity.xlsx")
N <- as.table(cbind(c(Activity$Cathemeral), c(Activity$Diurnal),c(Activity$Nocturnal)))
dimnames(N) <- list(Taxa = c(Activity$Taxa),  Activity = c("Cathemeral","Diurnal","Nocturnal"))
P<-N/rowSums(N)
P
library(graphics)
mosaicplot(N, shade = TRUE, las=1,main = "Hibernation proportion")


chisq<-chisq.test(N)
chisq
chisq$residuals
library(chisq.posthoc.test)
chisq.posthoc.test(N)


#### Avec les sous ordres
chisq<-chisq.test(N[2:3,])
chisq
chisq$residuals
library(chisq.posthoc.test)
chisq.posthoc.test(N[2:3,])

#### Avec les familles
chisq<-chisq.test(N[4:6,])
chisq
chisq$residuals
library(chisq.posthoc.test)
chisq.posthoc.test(N[4:6,])

library(graphics)
mosaicplot(N, shade = TRUE, las=1,main = "Activity proportion")


############################################### Test of variance
```

```
## Social type

# Carnivora
Carni_Social_type<-Ecology_complete_S02bis$Social_type
Carni_Social_type<-table(Ecology_complete_S02bis$Social_type)

# Caniformia
Canif_data=droplevels(Ecology_complete_S02bis[Ecology_complete_S02bis$Suborder=="Caniformia",c(AllVarEQ_vulnerability,"Species")])
Canif_CompData2=comparative.data(PhyloTree,data=Canif_data[,c(AllVarEQ_vulnerability,"Species")],names.col="Species")
Caniformia_Social_type<-Canif_CompData2$data$Social_type
Caniformia_Social_type<-table(Canif_CompData2$data$Social_type)
# Feliformia
Felif_data=droplevels(Ecology_complete_S02bis[Ecology_complete_S02bis$Suborder=="Feliformia",c(AllVarEQ_vulnerability,"Species")])
Felif_CompData2=comparative.data(PhyloTree,data=Felif_data[,c(AllVarEQ_vulnerability,"Species")],names.col="Species")
Feliformia_Social_type<-Felif_CompData2$data$Social_type
Feliformia_Social_type<-table(Felif_CompData2$data$Social_type)
# Canidae
Canid_data=droplevels(Ecology_complete_S02bis[Ecology_complete_S02bis$Family=="Canidae",c(AllVarEQ_vulnerability,"Species")])
Canid_CompData2=comparative.data(PhyloTreePrunned,data=Canid_data[,c(AllVarEQ_vulnerability,"Species")],names.col="Species")
Canidae_Social_type<-Canid_CompData2$data$Social_type
Canidae_Social_type<-table(Canid_CompData2$data$Social_type)
# Felidae
Felid_data=droplevels(Ecology_complete_S02bis[Ecology_complete_S02bis$Family=="Felidae",c(AllVarEQ_vulnerability,"Species")])
Felid_CompData2=comparative.data(PhyloTree,data=Felid_data[,c(AllVarEQ_vulnerability,"Species")],names.col="Species")
Felidae_Social_type<-Felid_CompData2$data$Social_type
Felidae_Social_type<-table(Felid_CompData2$data$Social_type)
#Mustelidae
Must_data=droplevels(Ecology_complete_S02bis[Ecology_complete_S02bis$Family=="Mustelidae",c(AllVarEQ_vulnerability,"Species")])
Must_CompData2=comparative.data(PhyloTree,data=Must_data[,c(AllVarEQ_vulnerability,"Species")],names.col="Species")
Mustelidae_Social_type<-Must_CompData2$data$Social_type
Mustelidae_Social_type<-table(Must_CompData2$data$Social_type)


#### Avec tous les taxa
Social_type <- read_excel("C:/Users/mmar3/Dropbox/Dossier pro/Projet FYSSEN/Article - Brain mass/Matrix/Var_Social_type.xlsx")
N <- as.table(cbind(c(Social_type$a0),
c(Social_type$a1),c(Social_type$a2),c(Social_type$a3),c(Social_type$a4),c(Social_type$a5),c(Social_type$a6)))
dimnames(N) <- list(Taxa = c(Social_type$Taxa),  Social_type = c("a0","a1","a2","a3","a4","a5","a6"))
P<-N/rowSums(N)
```

```
P
library(graphics)
mosaicplot(N, shade = TRUE, las=1,main = "Hibernation proportion")


chisq<-chisq.test(N)
chisq
chisq$residuals
library(chisq.posthoc.test)
chisq.posthoc.test(N)


#### Avec les sous ordres
chisq<-chisq.test(N[2:3,])
chisq
chisq$residuals
library(chisq.posthoc.test)
chisq.posthoc.test(N[2:3,])

#### Avec les familles
Social_type <- read_excel("C:/Users/mmar3/Dropbox/Dossier pro/Projet FYSSEN/Article - Brain mass/Matrix/Var_Social_type2.xlsx")
N <- as.table(cbind(c(Social_type$a0),
c(Social_type$a1),c(Social_type$a2),c(Social_type$a3),c(Social_type$a4),c(Social_type$a5)))
dimnames(N) <- list(Taxa = c(Social_type$Taxa),  Social_type = c("a0","a1","a2","a3","a4","a5"))
P<-N/rowSums(N)
chisq<-chisq.test(N)
chisq
chisq$residuals
library(chisq.posthoc.test)
chisq.posthoc.test(N)

library(graphics)
mosaicplot(N, shade = TRUE, las=1,main = "Social_type proportion")









############### Home range

library(ggpubr)
Car <- c(Ecology_complete_S02bis$LogHome_range)
Canif <- c(Canif_data$LogHome_range)
Felif <- c(Felif_data$LogHome_range)
```

```
Canid <- c(Canid_data$LogHome_range)
Felid <- c(Felid_data$LogHome_range)
Must <- c(Must_data$LogHome_range)

#Color palette (dans l'ordre alphabétique)
# Canidae #FF0000
# Caniformia #99FFFF
# Carnivora  #000000
# Felidae #FF9900
# Feliformia #FF3399
# Mustelidae #33FFFF

# Calcul de la variation
var(Car)
var(Canif)
var(Felif)
var(Canid)
var(Felid)
var(Must)

#### Tous les taxons
Data <- data.frame(Y=c(Car, Canif, Felif, Canid, Felid, Must),Taxa =factor(rep(c("Carinvora", "Caniformia", "Feliformia", "Canidae","Felidae","Mustelidae"),  times=c(length(Car), length(Canif), length(Felif), length(Canid),length(Felid),length(Must)))))
fm1 <- aov(Y~Taxa, data=Data)
anova(fm1)
TukeyHSD(fm1)
ggdensity(Data, x = "Y",add = "mean", color = "Taxa", fill=NA, size=1, palette = c("#FF0000","#99FF99","#000000","#FF9900","#FF3399","#33FFFF"))
bartlett.test(Data$Y,Data$Taxa)
#Variance Carnivora
Data <- data.frame(Y=c(Car),Taxa =factor(rep(c("Carinvora"),  times=c(length(Car)))))
var(Data$Y)
#Variance Caniformia
Data <- data.frame(Y=c(Canif),Taxa =factor(rep(c("Caniformia"),  times=c(length(Canif)))))
var(Data$Y)
#Variance Feliformia
Data <- data.frame(Y=c(Felif),Taxa =factor(rep(c("Feliformia"),  times=c(length(Felif)))))
var(Data$Y)
#Variance Canidae
Data <- data.frame(Y=c(Canid),Taxa =factor(rep(c("Canidae"),  times=c(length(Canid)))))
var(Data$Y)
#Variance Felidae
Data <- data.frame(Y=c(Felid),Taxa =factor(rep(c("Felidae"),  times=c(length(Felid)))))
var(Data$Y)
#Variance Mustelidae
Data <- data.frame(Y=c(Must),Taxa =factor(rep(c("Mustelidae"),  times=c(length(Must)))))
var(Data$Y)



### Entre les sous ordres
```

```
Data <- data.frame(Y=c(Canif, Felif),Taxa =factor(rep(c( "Caniformia", "Feliformia"),
times=c(length(Canif), length(Felif)))))
fm1 <- aov(Y~Taxa, data=Data)
anova(fm1)
TukeyHSD(fm1)
ggdensity(Data, x = "Y",add = "mean",color = "Taxa", fill = NA, size=1,  palette =
c("#99FFFF","#FF3399"))
bartlett.test(Data$Y,Data$Taxa)

### Entre les familles
Data <- data.frame(Y=c(Canid, Felid, Must),Taxa =factor(rep(c("Canidae","Felidae","Mustelidae"),
times=c(length(Canid),length(Felid),length(Must)))))
fm1 <- aov(Y~Taxa, data=Data)
anova(fm1)
TukeyHSD(fm1)
ggdensity(Data, x = "Y",add = "mean",color = "Taxa", fill = NA, size=1, palette =
c("#FF0000","#FF9900","#33FFFF"))
bartlett.test(Data$Y,Data$Taxa)




############### Geograpgic range

library(ggpubr)
Car <- c(Ecology_complete_S02bis$LogGeographic_range)
Canif <- c(Canif_data$LogGeographic_range)
Felif <- c(Felif_data$LogGeographic_range)
Canid <- c(Canid_data$LogGeographic_range)
Felid <- c(Felid_data$LogGeographic_range)
Must <- c(Must_data$LogGeographic_range)

#Color palette (dans l'ordre alphabétique)
# Canidae #FF0000
# Caniformia #99FFFF
# Carnivora  #000000
# Felidae #FF9900
# Feliformia #FF3399
# Mustelidae #33FFFF

# Calcul de la variation
var(Car)
var(Canif)
var(Felif)
var(Canid)
var(Felid)
var(Must)

#### Tous les taxons
Data <- data.frame(Y=c(Car, Canif, Felif, Canid, Felid, Must),Taxa =factor(rep(c("Carinvora",
"Caniformia", "Feliformia", "Canidae","Felidae","Mustelidae"),  times=c(length(Car), length(Canif),
length(Felif), length(Canid),length(Felid),length(Must)))))
```

```
fm1 <- aov(Y~Taxa, data=Data)
anova(fm1)
TukeyHSD(fm1)
ggdensity(Data, x = "Y",add = "mean", color = "Taxa", fill=NA, size=1, palette =
c("#FF0000","#99FF99","#000000","#FF9900","#FF3399","#33FFFF"))
bartlett.test(Data$Y,Data$Taxa)
#Variance Carnivora
Data <- data.frame(Y=c(Car),Taxa =factor(rep(c("Carinvora"),  times=c(length(Car)))))
var(Data$Y)
#Variance Caniformia
Data <- data.frame(Y=c(Canif),Taxa =factor(rep(c("Caniformia"),  times=c(length(Canif)))))
var(Data$Y)
#Variance Feliformia
Data <- data.frame(Y=c(Felif),Taxa =factor(rep(c("Feliformia"),  times=c(length(Felif)))))
var(Data$Y)
#Variance Canidae
Data <- data.frame(Y=c(Canid),Taxa =factor(rep(c("Canidae"),  times=c(length(Canid)))))
var(Data$Y)
#Variance Felidae
Data <- data.frame(Y=c(Felid),Taxa =factor(rep(c("Felidae"),  times=c(length(Felid)))))
var(Data$Y)
#Variance Mustelidae
Data <- data.frame(Y=c(Must),Taxa =factor(rep(c("Mustelidae"),  times=c(length(Must)))))
var(Data$Y)


### Entre les sous ordres
Data <- data.frame(Y=c(Canif, Felif),Taxa =factor(rep(c( "Caniformia", "Feliformia"),
times=c(length(Canif), length(Felif)))))
fm1 <- aov(Y~Taxa, data=Data)
anova(fm1)
TukeyHSD(fm1)
ggdensity(Data, x = "Y",add = "mean",color = "Taxa", fill = NA, size=1,  palette =
c("#99FFFF","#FF3399"))
bartlett.test(Data$Y,Data$Taxa)

### Entre les familles
Data <- data.frame(Y=c(Canid, Felid, Must),Taxa =factor(rep(c("Canidae","Felidae","Mustelidae"),
times=c(length(Canid),length(Felid),length(Must)))))
fm1 <- aov(Y~Taxa, data=Data)
anova(fm1)
TukeyHSD(fm1)
ggdensity(Data, x = "Y",add = "mean",color = "Taxa", fill = NA, size=1, palette =
c("#FF0000","#FF9900","#33FFFF"))
bartlett.test(Data$Y,Data$Taxa)



############### Group size

library(ggpubr)
```

```
Car <- log(c(Ecology_complete_S02bis$Group_size))
Canif <- log(c(Canif_data$Group_size))
Felif <- log(c(Felif_data$Group_size))
Canid <- log(c(Canid_data$Group_size))
Felid <- log(c(Felid_data$Group_size))
Must <- log(c(Must_data$Group_size))

#Color palette (dans l'ordre alphabétique)
# Canidae #FF0000
# Caniformia #99FFFF
# Carnivora  #000000
# Felidae #FF9900
# Feliformia #FF3399
# Mustelidae #33FFFF

# Calcul de la variation
var(Car)
var(Canif)
var(Felif)
var(Canid)
var(Felid)
var(Must)

#### Tous les taxons
Data <- data.frame(Y=c(Car, Canif, Felif, Canid, Felid, Must),Taxa =factor(rep(c("Carinvora",
"Caniformia", "Feliformia", "Canidae","Felidae","Mustelidae"),  times=c(length(Car), length(Canif),
length(Felif), length(Canid),length(Felid),length(Must)))))
fm1 <- aov(Y~Taxa, data=Data)
anova(fm1)
TukeyHSD(fm1)
ggdensity(Data, x = "Y",add = "mean", color = "Taxa", fill=NA, size=1, palette =
c("#FF0000","#99FF99","#000000","#FF9900","#FF3399","#33FFFF"))
bartlett.test(Data$Y,Data$Taxa)
#Variance Carnivora
Data <- data.frame(Y=c(Car),Taxa =factor(rep(c("Carinvora"),  times=c(length(Car)))))
var(Data$Y)
#Variance Caniformia
Data <- data.frame(Y=c(Canif),Taxa =factor(rep(c("Caniformia"),  times=c(length(Canif)))))
var(Data$Y)
#Variance Feliformia
Data <- data.frame(Y=c(Felif),Taxa =factor(rep(c("Feliformia"),  times=c(length(Felif)))))
var(Data$Y)
#Variance Canidae
Data <- data.frame(Y=c(Canid),Taxa =factor(rep(c("Canidae"),  times=c(length(Canid)))))
var(Data$Y)
#Variance Felidae
Data <- data.frame(Y=c(Felid),Taxa =factor(rep(c("Felidae"),  times=c(length(Felid)))))
var(Data$Y)
#Variance Mustelidae
Data <- data.frame(Y=c(Must),Taxa =factor(rep(c("Mustelidae"),  times=c(length(Must)))))
var(Data$Y)
```

```
### Entre les sous ordres
Data <- data.frame(Y=c(Canif, Felif),Taxa =factor(rep(c( "Caniformia", "Feliformia"),
times=c(length(Canif), length(Felif)))))
fm1 <- aov(Y~Taxa, data=Data)
anova(fm1)
TukeyHSD(fm1)
ggdensity(Data, x = "Y",add = "mean",color = "Taxa", fill = NA, size=1,  palette =
c("#99FFFF","#FF3399"))
bartlett.test(Data$Y,Data$Taxa)

### Entre les familles
Data <- data.frame(Y=c(Canid, Felid, Must),Taxa =factor(rep(c("Canidae","Felidae","Mustelidae"),
times=c(length(Canid),length(Felid),length(Must)))))
fm1 <- aov(Y~Taxa, data=Data)
anova(fm1)
TukeyHSD(fm1)
ggdensity(Data, x = "Y",add = "mean",color = "Taxa", fill = NA, size=1, palette =
c("#FF0000","#FF9900","#33FFFF"))
bartlett.test(Data$Y,Data$Taxa)



############### Gestation

library(ggpubr)
Car <- log(c(Ecology_complete_S02bis$Gestation))
Canif <- log(c(Canif_data$Gestation))
Felif <- log(c(Felif_data$Gestation))
Canid <- log(c(Canid_data$Gestation))
Felid <- log(c(Felid_data$Gestation))
Must <- log(c(Must_data$Gestation))

#Color palette (dans l'ordre alphabétique)
# Canidae #FF0000
# Caniformia #99FFFF
# Carnivora  #000000
# Felidae #FF9900
# Feliformia #FF3399
# Mustelidae #33FFFF

# Calcul de la variation
var(Car)
var(Canif)
var(Felif)
var(Canid)
var(Felid)
var(Must)

#### Tous les taxons
Data <- data.frame(Y=c(Car, Canif, Felif, Canid, Felid, Must),Taxa =factor(rep(c("Carinvora",
"Caniformia", "Feliformia", "Canidae","Felidae","Mustelidae"),  times=c(length(Car), length(Canif),
length(Felif), length(Canid),length(Felid),length(Must)))))
```

```
fm1 <- aov(Y~Taxa, data=Data)
anova(fm1)
TukeyHSD(fm1)
ggdensity(Data, x = "Y",add = "mean", color = "Taxa", fill=NA, size=1, palette =
c("#FF0000","#99FF99","#000000","#FF9900","#FF3399","#33FFFF"))
bartlett.test(Data$Y,Data$Taxa)
#Variance Carnivora
Data <- data.frame(Y=c(Car),Taxa =factor(rep(c("Carinvora"),  times=c(length(Car)))))
var(Data$Y)
#Variance Caniformia
Data <- data.frame(Y=c(Canif),Taxa =factor(rep(c("Caniformia"),  times=c(length(Canif)))))
var(Data$Y)
#Variance Feliformia
Data <- data.frame(Y=c(Felif),Taxa =factor(rep(c("Feliformia"),  times=c(length(Felif)))))
var(Data$Y)
#Variance Canidae
Data <- data.frame(Y=c(Canid),Taxa =factor(rep(c("Canidae"),  times=c(length(Canid)))))
var(Data$Y)
#Variance Felidae
Data <- data.frame(Y=c(Felid),Taxa =factor(rep(c("Felidae"),  times=c(length(Felid)))))
var(Data$Y)
#Variance Mustelidae
Data <- data.frame(Y=c(Must),Taxa =factor(rep(c("Mustelidae"),  times=c(length(Must)))))
var(Data$Y)

### Entre les sous ordres
Data <- data.frame(Y=c(Canif, Felif),Taxa =factor(rep(c( "Caniformia", "Feliformia"),
times=c(length(Canif), length(Felif)))))
fm1 <- aov(Y~Taxa, data=Data)
anova(fm1)
TukeyHSD(fm1)
ggdensity(Data, x = "Y",add = "mean",color = "Taxa", fill = NA, size=1,  palette =
c("#99FFFF","#FF3399"))
bartlett.test(Data$Y,Data$Taxa)

### Entre les familles
Data <- data.frame(Y=c(Canid, Felid, Must),Taxa =factor(rep(c("Canidae","Felidae","Mustelidae"),
times=c(length(Canid),length(Felid),length(Must)))))
fm1 <- aov(Y~Taxa, data=Data)
anova(fm1)
TukeyHSD(fm1)
ggdensity(Data, x = "Y",add = "mean",color = "Taxa", fill = NA, size=1, palette =
c("#FF0000","#FF9900","#33FFFF"))
bartlett.test(Data$Y,Data$Taxa)




############### Interbirth
```

```
library(ggpubr)
Car <- log(c(Ecology_complete_S02bis$Interbirth))
Canif <- log(c(Canif_data$Interbirth))
Felif <- log(c(Felif_data$Interbirth))
Canid <- log(c(Canid_data$Interbirth))
Felid <- log(c(Felid_data$Interbirth))
Must <- log(c(Must_data$Interbirth))

#Color palette (dans l'ordre alphabétique)
# Canidae #FF0000
# Caniformia #99FFFF
# Carnivora  #000000
# Felidae #FF9900
# Feliformia #FF3399
# Mustelidae #33FFFF

# Calcul de la variation
var(Car)
var(Canif)
var(Felif)
var(Canid)
var(Felid)
var(Must)

#### Tous les taxons
Data <- data.frame(Y=c(Car, Canif, Felif, Canid, Felid, Must),Taxa =factor(rep(c("Carinvora",
"Caniformia", "Feliformia", "Canidae","Felidae","Mustelidae"),  times=c(length(Car), length(Canif),
length(Felif), length(Canid),length(Felid),length(Must)))))
fm1 <- aov(Y~Taxa, data=Data)
anova(fm1)
TukeyHSD(fm1)
ggdensity(Data, x = "Y",add = "mean", color = "Taxa", fill=NA, size=1, palette =
c("#FF0000","#99FF99","#000000","#FF9900","#FF3399","#33FFFF"))
bartlett.test(Data$Y,Data$Taxa)
#Variance Carnivora
Data <- data.frame(Y=c(Car),Taxa =factor(rep(c("Carinvora"),  times=c(length(Car)))))
var(Data$Y)
#Variance Caniformia
Data <- data.frame(Y=c(Canif),Taxa =factor(rep(c("Caniformia"),  times=c(length(Canif)))))
var(Data$Y)
#Variance Feliformia
Data <- data.frame(Y=c(Felif),Taxa =factor(rep(c("Feliformia"),  times=c(length(Felif)))))
var(Data$Y)
#Variance Canidae
Data <- data.frame(Y=c(Canid),Taxa =factor(rep(c("Canidae"),  times=c(length(Canid)))))
var(Data$Y)
#Variance Felidae
Data <- data.frame(Y=c(Felid),Taxa =factor(rep(c("Felidae"),  times=c(length(Felid)))))
var(Data$Y)
#Variance Mustelidae
Data <- data.frame(Y=c(Must),Taxa =factor(rep(c("Mustelidae"),  times=c(length(Must)))))
var(Data$Y)
```

```
### Entre les sous ordres
Data <- data.frame(Y=c(Canif, Felif),Taxa =factor(rep(c( "Caniformia", "Feliformia"),
times=c(length(Canif), length(Felif)))))
fm1 <- aov(Y~Taxa, data=Data)
anova(fm1)
TukeyHSD(fm1)
ggdensity(Data, x = "Y",add = "mean",color = "Taxa", fill = NA, size=1,  palette =
c("#99FFFF","#FF3399"))
bartlett.test(Data$Y,Data$Taxa)

### Entre les familles
Data <- data.frame(Y=c(Canid, Felid, Must),Taxa =factor(rep(c("Canidae","Felidae","Mustelidae"),
times=c(length(Canid),length(Felid),length(Must)))))
fm1 <- aov(Y~Taxa, data=Data)
anova(fm1)
TukeyHSD(fm1)
ggdensity(Data, x = "Y",add = "mean",color = "Taxa", fill = NA, size=1, palette =
c("#FF0000","#FF9900","#33FFFF"))
bartlett.test(Data$Y,Data$Taxa)




############### litter_size

library(ggpubr)
Car <- log(c(Ecology_complete_S02bis$Litter_size))
Canif <- log(c(Canif_data$Litter_size))
Felif <- log(c(Felif_data$Litter_size))
Canid <- log(c(Canid_data$Litter_size))
Felid <- log(c(Felid_data$Litter_size))
Must <- log(c(Must_data$Litter_size))

#Color palette (dans l'ordre alphabétique)
# Canidae #FF0000
# Caniformia #99FFFF
# Carnivora  #000000
# Felidae #FF9900
# Feliformia #FF3399
# Mustelidae #33FFFF

# Calcul de la variation
var(Car)
var(Canif)
var(Felif)
var(Canid)
var(Felid)
var(Must)

#### Tous les taxons
```

```
Data <- data.frame(Y=c(Car, Canif, Felif, Canid, Felid, Must),Taxa =factor(rep(c("Carinvora",
"Caniformia", "Feliformia", "Canidae","Felidae","Mustelidae"),  times=c(length(Car), length(Canif),
length(Felif), length(Canid),length(Felid),length(Must)))))
fm1 <- aov(Y~Taxa, data=Data)
anova(fm1)
TukeyHSD(fm1)
ggdensity(Data, x = "Y",add = "mean", color = "Taxa", fill=NA, size=1, palette =
c("#FF0000","#99FF99","#000000","#FF9900","#FF3399","#33FFFF"))
bartlett.test(Data$Y,Data$Taxa)
#Variance Carnivora
Data <- data.frame(Y=c(Car),Taxa =factor(rep(c("Carinvora"),  times=c(length(Car)))))
var(Data$Y)
#Variance Caniformia
Data <- data.frame(Y=c(Canif),Taxa =factor(rep(c("Caniformia"),  times=c(length(Canif)))))
var(Data$Y)
#Variance Feliformia
Data <- data.frame(Y=c(Felif),Taxa =factor(rep(c("Feliformia"),  times=c(length(Felif)))))
var(Data$Y)
#Variance Canidae
Data <- data.frame(Y=c(Canid),Taxa =factor(rep(c("Canidae"),  times=c(length(Canid)))))
var(Data$Y)
#Variance Felidae
Data <- data.frame(Y=c(Felid),Taxa =factor(rep(c("Felidae"),  times=c(length(Felid)))))
var(Data$Y)
#Variance Mustelidae
Data <- data.frame(Y=c(Must),Taxa =factor(rep(c("Mustelidae"),  times=c(length(Must)))))
var(Data$Y)

### Entre les sous ordres
Data <- data.frame(Y=c(Canif, Felif),Taxa =factor(rep(c( "Caniformia", "Feliformia"),
times=c(length(Canif), length(Felif)))))
fm1 <- aov(Y~Taxa, data=Data)
anova(fm1)
TukeyHSD(fm1)
ggdensity(Data, x = "Y",add = "mean",color = "Taxa", fill = NA, size=1,  palette =
c("#99FFFF","#FF3399"))
bartlett.test(Data$Y,Data$Taxa)

### Entre les familles
Data <- data.frame(Y=c(Canid, Felid, Must),Taxa =factor(rep(c("Canidae","Felidae","Mustelidae"),
times=c(length(Canid),length(Felid),length(Must)))))
fm1 <- aov(Y~Taxa, data=Data)
anova(fm1)
TukeyHSD(fm1)
ggdensity(Data, x = "Y",add = "mean",color = "Taxa", fill = NA, size=1, palette =
c("#FF0000","#FF9900","#33FFFF"))
bartlett.test(Data$Y,Data$Taxa)
```

```
############### Weanning

library(ggpubr)
Car <- log(c(Ecology_complete_S02bis$Weaning))
Canif <- log(c(Canif_data$Weaning))
Felif <- log(c(Felif_data$Weaning))
Canid <- log(c(Canid_data$Weaning))
Felid <- log(c(Felid_data$Weaning))
Must <- log(c(Must_data$Weaning))

#Color palette (dans l'ordre alphabétique)
# Canidae #FF0000
# Caniformia #99FFFF
# Carnivora  #000000
# Felidae #FF9900
# Feliformia #FF3399
# Mustelidae #33FFFF

# Calcul de la variation
var(Car)
var(Canif)
var(Felif)
var(Canid)
var(Felid)
var(Must)

#### Tous les taxons
Data <- data.frame(Y=c(Car, Canif, Felif, Canid, Felid, Must),Taxa =factor(rep(c("Carinvora",
"Caniformia", "Feliformia", "Canidae","Felidae","Mustelidae"),  times=c(length(Car), length(Canif),
length(Felif), length(Canid),length(Felid),length(Must)))))
fm1 <- aov(Y~Taxa, data=Data)
anova(fm1)
TukeyHSD(fm1)
ggdensity(Data, x = "Y",add = "mean", color = "Taxa", fill=NA, size=1, palette =
c("#FF0000","#99FF99","#000000","#FF9900","#FF3399","#33FFFF"))
bartlett.test(Data$Y,Data$Taxa)
#Variance Carnivora
Data <- data.frame(Y=c(Car),Taxa =factor(rep(c("Carinvora"),  times=c(length(Car)))))
var(Data$Y)
#Variance Caniformia
Data <- data.frame(Y=c(Canif),Taxa =factor(rep(c("Caniformia"),  times=c(length(Canif)))))
var(Data$Y)
#Variance Feliformia
Data <- data.frame(Y=c(Felif),Taxa =factor(rep(c("Feliformia"),  times=c(length(Felif)))))
var(Data$Y)
#Variance Canidae
Data <- data.frame(Y=c(Canid),Taxa =factor(rep(c("Canidae"),  times=c(length(Canid)))))
var(Data$Y)
#Variance Felidae
Data <- data.frame(Y=c(Felid),Taxa =factor(rep(c("Felidae"),  times=c(length(Felid)))))
var(Data$Y)
#Variance Mustelidae
```

```
Data <- data.frame(Y=c(Must),Taxa =factor(rep(c("Mustelidae"),  times=c(length(Must)))))
var(Data$Y)

### Entre les sous ordres
Data <- data.frame(Y=c(Canif, Felif),Taxa =factor(rep(c( "Caniformia", "Feliformia"),
times=c(length(Canif), length(Felif)))))
fm1 <- aov(Y~Taxa, data=Data)
anova(fm1)
TukeyHSD(fm1)
ggdensity(Data, x = "Y",add = "mean",color = "Taxa", fill = NA, size=1,  palette =
c("#99FFFF","#FF3399"))
bartlett.test(Data$Y,Data$Taxa)

### Entre les familles
Data <- data.frame(Y=c(Canid, Felid, Must),Taxa =factor(rep(c("Canidae","Felidae","Mustelidae"),
times=c(length(Canid),length(Felid),length(Must)))))
fm1 <- aov(Y~Taxa, data=Data)
anova(fm1)
TukeyHSD(fm1)
ggdensity(Data, x = "Y",add = "mean",color = "Taxa", fill = NA, size=1, palette =
c("#FF0000","#FF9900","#33FFFF"))
bartlett.test(Data$Y,Data$Taxa)





############### Temperature

Ecology_complete_S02$TempK<-Ecology_complete_S02$Temperature+273.15

library(ggpubr)
Car <- log(c(Ecology_complete_S02$TempK))
Canif <- log(c(Canif_data$TempK))
Felif <- log(c(Felif_data$TempK))
Canid <- log(c(Canid_data$TempK))
Felid <- log(c(Felid_data$TempK))
Must <- log(c(Must_data$TempK))

#Color palette (dans l'ordre alphabétique)
# Canidae #FF0000
# Caniformia #99FFFF
# Carnivora  #000000
# Felidae #FF9900
# Feliformia #FF3399
# Mustelidae #33FFFF

# Calcul de la variation
var(Car)
var(Canif)
var(Felif)
```

```
var(Canid)
var(Felid)
var(Must)

#### Tous les taxons
Data <- data.frame(Y=c(Car, Canif, Felif, Canid, Felid, Must),Taxa =factor(rep(c("Carinvora",
"Caniformia", "Feliformia", "Canidae","Felidae","Mustelidae"),  times=c(length(Car), length(Canif),
length(Felif), length(Canid),length(Felid),length(Must)))))
fm1 <- aov(Y~Taxa, data=Data)
anova(fm1)
TukeyHSD(fm1)
ggdensity(Data, x = "Y",add = "mean", color = "Taxa", fill=NA, size=1, palette =
c("#FF0000","#99FF99","#000000","#FF9900","#FF3399","#33FFFF"))
bartlett.test(Data$Y,Data$Taxa)
#Variance Carnivora
Data <- data.frame(Y=c(Car),Taxa =factor(rep(c("Carinvora"),  times=c(length(Car)))))
var(Data$Y)
#Variance Caniformia
Data <- data.frame(Y=c(Canif),Taxa =factor(rep(c("Caniformia"),  times=c(length(Canif)))))
var(Data$Y)
#Variance Feliformia
Data <- data.frame(Y=c(Felif),Taxa =factor(rep(c("Feliformia"),  times=c(length(Felif)))))
var(Data$Y)
#Variance Canidae
Data <- data.frame(Y=c(Canid),Taxa =factor(rep(c("Canidae"),  times=c(length(Canid)))))
var(Data$Y)
#Variance Felidae
Data <- data.frame(Y=c(Felid),Taxa =factor(rep(c("Felidae"),  times=c(length(Felid)))))
var(Data$Y)
#Variance Mustelidae
Data <- data.frame(Y=c(Must),Taxa =factor(rep(c("Mustelidae"),  times=c(length(Must)))))
var(Data$Y)

### Entre les sous ordres
Data <- data.frame(Y=c(Canif, Felif),Taxa =factor(rep(c( "Caniformia", "Feliformia"),
times=c(length(Canif), length(Felif)))))
fm1 <- aov(Y~Taxa, data=Data)
anova(fm1)
TukeyHSD(fm1)
ggdensity(Data, x = "Y",add = "mean",color = "Taxa", fill = NA, size=1,  palette =
c("#99FFFF","#FF3399"))
bartlett.test(Data$Y,Data$Taxa)

### Entre les familles
Data <- data.frame(Y=c(Canid, Felid, Must),Taxa =factor(rep(c("Canidae","Felidae","Mustelidae"),
times=c(length(Canid),length(Felid),length(Must)))))
fm1 <- aov(Y~Taxa, data=Data)
anova(fm1)
TukeyHSD(fm1)
ggdensity(Data, x = "Y",add = "mean",color = "Taxa", fill = NA, size=1, palette =
c("#FF0000","#FF9900","#33FFFF"))
bartlett.test(Data$Y,Data$Taxa)
```
