# Supplementary material for: The impact of environmental factors on the evolution of brain size in carnivorans
Source: Commun Biol. 2022 Sep 21;5:998. doi: 10.1038/s42003-022-03748-4 (PMC9492690; doi:10.1038/s42003-022-03748-4)
Supplement: Supplementary file 6 — Reporting Summary [file 42003_2022_3748_MOESM6_ESM.pdf]

Corresponding author(s): Margot Michaud
Last updated by author(s): Jun 20, 2022

Reporting Summary

Nature Portfolio wishes to improve the reproducibility of the work that we publish. This form provides structure for consistency and transparency in reporting. For further information on Nature Portfolio policies, see our Editorial Policies and the Editorial Policy Checklist.

Statistics

For all statistical analyses, confirm that the following items are present in the figure legend, table legend, main text, or Methods section.

n/a	Confirmed	
☐	☒	The exact sample size (n) for each experimental group/condition, given as a discrete number and unit of measurement
☐	☒	A statement on whether measurements were taken from distinct samples or whether the same sample was measured repeatedly
☐	☒	The statistical test(s) used AND whether they are one- or two-sided<br>*Only common tests should be described solely by name; describe more complex techniques in the Methods section.*
☒	☐	A description of all covariates tested
☐	☒	A description of any assumptions or corrections, such as tests of normality and adjustment for multiple comparisons
☐	☒	A full description of the statistical parameters including central tendency (e.g. means) or other basic estimates (e.g. regression coefficient) AND variation (e.g. standard deviation) or associated estimates of uncertainty (e.g. confidence intervals)
☐	☒	For null hypothesis testing, the test statistic (e.g. F, t, r) with confidence intervals, effect sizes, degrees of freedom and P value noted<br>*Give P values as exact values whenever suitable.*
☐	☒	For Bayesian analysis, information on the choice of priors and Markov chain Monte Carlo settings
☐	☒	For hierarchical and complex designs, identification of the appropriate level for tests and full reporting of outcomes
☐	☒	Estimates of effect sizes (e.g. Cohen's d, Pearson's r), indicating how they were calculated

Our web collection on statistics for biologists contains articles on many of the points above.

Software and code

Policy information about availability of computer code

Data collection	All data are provided in the Supplementary Data
Data analysis	All software and R packages used in our study are described in the manuscript. The R code for this study will be made available on an online repository prior to publication.

For manuscripts utilizing custom algorithms or software that are central to the research but not yet described in published literature, software must be made available to editors and reviewers. We strongly encourage code deposition in a community repository (e.g. GitHub). See the Nature Portfolio guidelines for submitting code & software for further information.

Data

Policy information about availability of data

All manuscripts must include a data availability statement. This statement should provide the following information, where applicable:

- Accession codes, unique identifiers, or web links for publicly available datasets
- A description of any restrictions on data availability
- For clinical datasets or third party data, please ensure that the statement adheres to our policy

Data used in this study are available in the Supplementary information

Field-specific reporting

Please select the one below that is the best fit for your research. If you are not sure, read the appropriate sections before making your selection.

☐ Life sciences ☐ Behavioural & social sciences ☒ Ecological, evolutionary & environmental sciences

For a reference copy of the document with all sections, see nature.com/documents/nr-reporting-summary-flat.pdf

Ecological, evolutionary & environmental sciences study design

All studies must disclose on these points even when the disclosure is negative.

Study description	We studied the patterns and tempo of brain evolution within the order Carnivora and present the first large-scale comparative analysis of the effect of ecological, environmental, social, and physiological variables on relative brain size evolution. While controlling for phylogenetic history, we further test for body-brain allometric patterns and search for possible shifts in the carnivoran brain size evolution through time.
Research sample	We sampled 361 crania of museum specimens belonging to 158 terrestrial carnivoran species. All specimens were adults, preferably of wild origin, and we sampled both males and females when possible.
Sampling strategy	We sampled as many species as possible (i.e. 72% of the taxonomic diversity for this group) and included several individuals per species when possible to account for intra-specific variability (1 to 11 individuals)
Data collection	M.M. made the measurements on museum specimens previously scanned in 3D.
Timing and spatial scale	The data were collected between April and August 2020
Data exclusions	No data were excluded from our analyses
Reproducibility	All measurements taken on the specimens are provided in the Supplementary Data.
Randomization	There is no randomization in our study
Blinding	There is no blinding in our study

Did the study involve field work? ☐ Yes ☒ No

Reporting for specific materials, systems and methods

We require information from authors about some types of materials, experimental systems and methods used in many studies. Here, indicate whether each material, system or method listed is relevant to your study. If you are not sure if a list item applies to your research, read the appropriate section before selecting a response.

Materials & experimental systems

n/a	Involved in the study
☒	☐ Antibodies
☒	☐ Eukaryotic cell lines
☒	☐ Palaeontology and archaeology
☒	☐ Animals and other organisms
☒	☐ Human research participants
☒	☐ Clinical data
☒	☐ Dual use research of concern

Methods

n/a	Involved in the study
☒	☐ ChIP-seq
☒	☐ Flow cytometry
☒	☐ MRI-based neuroimaging
